# The cellular signaling crosstalk between memory B cells and tumor cells in nasopharyngeal carcinoma cannot be overlooked: Their involvement in tumor progression and treatment strategy is significant

**DOI:** 10.7150/jca.101420

**Published:** 2025-01-01

**Authors:** Haiyu Li, Yanjie Bian, Zhikai Xiahou, Zhijie Zhao, Fu Zhao, Qinghan Zhang

**Affiliations:** 1 Department of Otolaryngology, Songjiang Hospital Affiliated to Shanghai Jiao Tong University School of Medicine, Shanghai, China.; 2 Department of Clinical Laboratory, Songjiang Hospital Affiliated to Shanghai Jiao Tong University School of Medicine, Shanghai, China.; 3 Shandong University of Traditional Chinese Medicine, Jinan, China.; 4 Xinxiang Medical University, Xinxiang, China.; 5 China Institute of Sport and Health Science, Beijing Sport University, Beijing, China.; 6 Department of Plastic and Reconstructive Surgery, Shanghai Ninth People's Hospital, School of Medicine, Shanghai Jiao Tong University, Shanghai, China.

**Keywords:** Molecular biomarkers, Cellular signaling network, Memory B cells, Immunotherapy, Nasopharyngeal carcinoma, Single-cell RNA sequencing

## Abstract

**Background:** Nasopharyngeal carcinoma (NPC) refers to a cancerous tumor that develops in the upper and side walls of the nasopharyngeal cavity. Typically, individuals are often diagnosed with the disease when it has already progressed significantly, and those with advanced NPC tend to have an unfavorable outlook in terms of response rate to targeted treatments and overall clinical survival. Various molecular mechanisms, including Myeloid-derived suppressor cells and factors like PD-L1, have been explored to enhance the outcome of NPC. However, there are still challenges to be addressed in terms of identifying symptoms at an early stage, making precise predictions about the chances of cancer returning and spreading, and devising successful approaches for treatment. The activation of B cells and their corresponding pathways holds potential for developing enhanced immune therapeutic strategies. Nevertheless, the comprehensive understanding of the intricate association between B cells and NPC tumor cells remains incomplete. Hence, this study employed single-cell multi-omics analysis to investigate the molecular biomarkers and prognostic factors linked to B cell subpopulations in human NPC while examining the underlying mechanisms.

**Materials and Methods:** The Gene Expression Omnibus database provided tumor and blood samples obtained from patients diagnosed with NPC. Subsequently, we analyzed these single-cell data. Following the assessment of NPC sample quality, we employed the R package 'Harmony' to mitigate batch discrepancies using PCA outcomes. The analysis of Gene Ontology, Gene Set Enrichment Analysis, and Kyoto Encyclopedia of Genes and Genomes was used to examine differentially expressed genes in B cell subpopulations of NPC tumors. The pseudo-temporal trajectories of B cells in NPC were studied using the Monocle and Slingshot software tools. In addition, the CellChat package was utilized to predict the incidence of intercellular communication between different subpopulations of B cells and cancerous cells. Furthermore, we utilized univariate Cox regression, LASSO, and multivariate Cox regression analysis to construct prognostic models. The immune cell infiltration was evaluated in tumor tissues using ESTIMATE, CIBERSORT, and xCell. Furthermore, the infercnv was employed to assess the extent of copy number variation in NPC cells. To forecast the potential reaction of particular tumor samples to chemotherapy, the R package called 'pRRophetic' was utilized.

**Results:** Single-cell RNA sequencing effectively identified various cell subgroups in NPC, including T/NK cells, B cells, plasma cells, myeloid cells, mast cells, and malignant cells. A comprehensive examination of the B cell subgroups revealed their division into 13 distinct groups, each with unique characteristics and functions. Enrichment analysis indicated that C4 CD86+ Memory B cells may play a role in inhibiting viral invasion and activity. Through trajectory analysis, we mapped the differentiation pathways of B cells and found that C4 CD86+ Memory B cells represent the final stage of this differentiation process. Furthermore, signal communication analysis revealed that C4 CD86+ Memory B cells have the potential to initiate interactions with malignant cells via the CD99-CD99, SEMA4-PLXNB2, and notably the CD46-JAG1 signaling pathways. To construct the CD86+ Memory B score, we employed univariate Cox regression analysis, LASSO regression analysis, and multivariate Cox regression analysis to screen 14 genes based on the top 100 marker genes of C4 CD86+ Memory B cells.

**Conclusion:** The results indicate that the C4 CD86+ Memory B cells may have a suppressive impact on viral activity in NPC. However, patients with a higher subgroup of CD86+Memory B scores exhibited a worse prognosis. This could be attributed to the crucial involvement of C4 CD86+ Memory B cells in the proliferation and differentiation of tumor cells, which occurs through the CD46-JAG1 signaling pathway. The discoveries provide significant insights into the fundamental mechanisms of developing NPC. Moreover, these factors greatly influence the prognosis of individuals suffering from this specific type of cancer and offer crucial perspectives for the advancement of future treatment approaches.

## Introduction

Nasopharyngeal carcinoma (NPC), a type of cancerous epithelial tumor, is found in the upper part and side of the nasopharyngeal cavity. It is more common in East and Southeast Asia 1-4. This type of cancer makes up around 30% of malignant tumors and 70-80% of head and neck tumors. Men are affected by it at a rate two to three times higher than women. Statistics indicate that in 2020, there were an estimated 133,354 new cases of NPC and 80,008 deaths worldwide. This considerable toll not only highlights the severity of the disease but also emphasizes the urgent need for effective prevention, early detection, and treatment strategies to mitigate its impact on global health and well-being 5,6. Apart from the presence of Epstein-Barr virus (EBV), recent studies have highlighted human papilloma virus (HPV) infection, alcohol consumption, smoking, and the intake of salt-preserved foods as notable risk factors 2. Presently, the prediction and selection of treatment alternatives for individuals diagnose with NPC primarily rely on the Tumor-lymph node-metastasis (TNM) staging system established by the Union for International Cancer Control and the American Joint Committee on Cancer (UICC/AJCC), 8th edition 7-10. However, due to the inherent heterogeneity of NPC tumor cells, NPC patients have significantly different survival outcomes at the same stage 11-13. More than 90% of patients diagnosed with NPC in its early stages can survive for 5 years, whereas the survival rate drops to 60% for patients in advanced stages 14. Typically, due to the concealed anatomical position of NPC and the absence of early signs or distinct clinical symptoms, the diagnosis of the illness is often postponed until it reaches advanced stages, with or without the spread to the cervical lymph nodes 15. Hence, a pressing requirement exists for a molecular biomarker that is both highly sensitive and specific, enabling early detection of the illness, forecasting outcomes, and anticipating the likelihood of relapse and spread 16.

Researchers and clinicians have extensively studied several processes associated with improved outcomes in NPC. They have employed advanced techniques such as genomics, transcriptomics, proteomics, and metabolomics, which have yielded numerous useful discoveries 17. Targets for immunotherapy in NPC include upregulated MDSC, PD-L1 overexpression, and various co-stimulatory factors linked to T-cell exhaustion and dysfunction. These factors encompass CTLA-4, LAG-3, TIGIT, TIM3, and CD276, which hinder the immune response and can be potential targets for immunotherapy 18. These studies have provided some ideas for the treatment of NPC. Nevertheless, until now, the emphasis of cancer immunotherapy has primarily been on T-cells, neglecting thorough investigation of other immune subgroups. This limited approach has hindered advancements in early detection, prognosis, and forecasting of NPC's recurrence and metastasis. Consequently, the current overall clinical survival rate for patients remains unsatisfactory. B lymphocytes are regarded as the primary cells responsible for humoral immunity. They enhance T cell reactions and hinder tumor advancement by both producing immunoglobulins and directly eliminating malignant cells. The properties play a significant role in the immune response against tumors within the tumor microenvironment (TME). Despite the advent of immune checkpoint inhibitors, not all individuals experience therapeutic benefits from these treatments, which specifically target immune responses associated with T-cells. The activation of the immune response through humoral immunity and the formation of tertiary lymphoid structure (TLS) are key functions of B cells and B cell-related pathways, including the CCL19, -21/CCR7 axis, and CXCL13/CXCR5 axis. play a key role in TME. However, they have some pro-tumorigenic effects in TME 19. Hence, it is imperative to acquire a more comprehensive comprehension of B cells and the pathways associated with them to devise efficient strategies for cancer management. Based on preclinical data, activation of B cells and B cell-associated pathways may open new opportunities for more effective immunotherapies, but we should be aware that they also have some pro-tumorigenic effects in TME. Enhanced comprehension regarding the control of B cells and pathways associated with B cells may open possibilities for novel approaches in the treatment of cancer 20. Hence, we employed single-cell RNA sequencing (scRNA-seq) data from Gene Expression Omnibus database (GEO) and bulk RNA sequencing (bulk RNA-seq) data from the Cancer Genome Atlas (TCGA) to investigate NPC, with a particular emphasis on examining molecular biomarkers and prognostic factors linked to B cell subpopulations, as well as exploring potential mechanisms. The research we conduct will establish a scientific foundation for enhanced NPC prevention, diagnosis, and treatment. Additionally, it is anticipated to expedite the advancement of personalized therapy, ultimately enhancing patients' prognosis and quality of life. Furthermore, our limited knowledge about the microenvironment of NPC consistently obstructs the improvement of therapeutic interventions. Hence, it is imperative to gain a deeper understanding of the composition of immune-infiltrating cells in NPC.

In general, numerous subgroups of tumors that infiltrate is being more and more acknowledged as potential indicators and targets for the early detection, prediction of future outcomes, and restoration of impaired immune system. Currently, multi-omics sequencing technologies have been widely applied in cancer research 21. Nevertheless, the past ten years have witnessed a dearth of comprehensive single-cell analysis methods, resulting in insufficient research on NPC. Our objective in this study was to offer a thorough and all-encompassing perspective on the diversity of tumors in NPC.

## Materials and Methods

### Data of single-cell RNA and processing

The single-cell RNA sequencing (scRNA-seq) data for NPC and peripheral blood lymphocyte samples (n=10) were obtained from GSE162025, accessible through the GEO database on the National Center for Biotechnology Information (NCBI) website (https://www.ncbi.nlm.nih.gov/geo/). The R software (version 4.2.0) and Seurat R package (version 4.3.0) were utilized to process the 10X genomics data from each sample 22,23.

### Data quality control and preprocessing

We performed quality control of the gene-cell data. Initially, we employed the DoubletFinder R package (version 2.0.3) 24 to detect and eliminate any duplicate cells that might have occurred due to cell encapsulation or cells that were not properly separated during sample preparation, as well as accidental merging of samples. In this study, we also excluded cells that expressed more than 25% of mitochondrial genes. We also excluded low-quality cells with fewer than 300 or more than 7500 genes detected, or fewer than 3 cells detected. Since we used data from publicly available databases, no ethical approval was required for this study.

### Removal of batch effects and clustering by scRNA-seq

The log(x+1) method was used to calculate the gene expression in each cell by multiplying the fraction of genes by 10,000 for the natural logarithmic transformation 25-27. Normalization was performed using NormalizedData 28,29, employing log normalization to adjust for differences in sequencing depth across samples This method transforms the raw count data into a format that is more suitable for downstream analysis by taking the logarithm of the counts after scaling. Subsequently, the top 2,000 highly variable genes were identified and filtered out using FindVariableFeatures 30, followed by the normalization process using the ScaleData normalization 31-33. These genes were then scaled before subjecting them to principal component analysis (PCA) 34,35. The R harmony R package (version 0.1.1) 36 was utilized to remove batch effects by analyzing the resulting components of PCA. By utilizing FindNeighbors for data coordination, we calculated the k closest neighbors and formed a shared nearest neighbor graph 37. To identify clusters, we utilized FindClusters to classify the data, followed by implementing the uniform manifold approximation and projection (UMAP) dimensionality reduction technique 38 to visually represent the identified clusters on a 2D map.

### Cell clustering analysis

To detect marker genes for each cluster, we employed the 'FindAllMarkers' function with parameters Threshold = 0.25, min.pct = 0.25, and min.diff.pct = 0.25. We used Seurat's DotPlot and featureplot tools to visualize the expression patterns of each marker gene in each cluster. To annotate the cell groups, we referred to known cellular markers and differentially expressed genes (DEGs) mentioned in the scientific literature. In addition, to explore the heterogeneity of B cells in NPC in more depth, we reclustered the cells in the B cell population. Each B cell subpopulation was annotated according to the expression of its different genes.

### Perform pathway enrichment analysis on DEGs using GO, KEGG, and GSEA

To analyze the functional enrichment of DEGs, including Gene Ontology (GO) 39-43 terms and Kyoto Encyclopedia of Genes and Genomes (KEGG) pathways 44-46, we utilized the R software's 'GOplot' package. During the analysis of GO, we assessed the enriched biological processes 47. The identification of crucial pathways and central genes was accomplished using the technique of gene set enrichment analysis (GSEA) 48. Enrichment analysis was performed to determine whether a set of a priori defined biological processes were enriched. Pathways were prioritized based on their normalized enrichment score, and only those with a *P-value* less than 0.05 were chosen for additional analysis. Gene entries for Gene Ontology Biological Process (GOBP) were used to perform GSEA enrichment analysis 49.

### Monocle trajectory analysis of B cell subpopulations

The Monocle R package (version 2.24.0) algorithm 50 was utilized to analyze cellular subtypes of B cells obtained from NPCs, enabling the construction of pseudo-temporal trajectories and identification of gene expression alterations during cell transitions. Monocle presents a technique called monoclonal trajectory analysis, utilizing an algorithm to comprehend the gene expression alterations that every cell must experience in a sequential manner as a component of a dynamic biological procedure, enabling us to observe these conditions in each individual cell. Each cell can be placed in the trajectory analysis based on its "trajectory" of gene expression changes. Cells were sorted along the trajectories and their trajectories were visualized in a reduced dimensional space.

### Slingshot pseudotemporal analysis of B cell subpopulations

The Slingshot package (version 2.6.0) 51,52 is utilized to infer cell lineages and identify approximate trajectory structures by applying minimum spanning trees to clusters through the getLineages function. Additionally, it estimates potential cell-level pseudotimes for each lineage using synchronized master curves fitted via the getCurves function. After trajectory inference, the relationship between gene expression levels and pseudotimes for each lineage was modeled using a Negative Binomial Generalized Additive Model (NB-GAM) via tradeSeq version 1.2.01. This approach employs smoothed spline curves to illustrate average gene expression levels as a function of pseudotime, capturing the continuous variation in gene expression across pseudotime.

We used Slingshot to perform pseudotemporal extrapolation of UMAP coordinate plots of B cell subpopulations to visualize pseudotemporal differentiation trajectories of B cell subpopulations and analyzed them in comparison to the pseudotemporal differentiation trajectories inferred by monocle2.

### Analysis of intercellular communication

To deducing and examining intercellular communication, we employed the CellChat R package (version 1.6.1) 53-56, an open database containing ligands, receptors, and their interactions, utilizing the default settings. To analyze cellular interaction, expression levels were computed in relation to the overall read-labeled profiles of the identical group of coding genes in every transcriptome. The average expression values were calculated for each individual cell cluster or cell sample.

### Construction and validation of risk models

Due to the fact that NPC is classified as a kind of head and neck squamous cell carcinoma (HNSCC), and since the TCGA database has more comprehensive gene expression data and clinical information for HNSCC patients, we utilized the data specifically from head and neck squamous carcinomas in the TCGA database. The expression values of genes were extracted and used to perform the univariate Cox regression analysis to screen for potential prognosis-related genes 57-59. Prognosis-related genes were chosen and genes with a p-value less than 0.05 were screened through univariate cox regression analysis 60,61. Prognostic models were then built using Least Absolute Shrinkage and Selection Operato (LASSO) regression (glmnet, version 4.1-6) with the least absolute shrinkage and selection operator. We constructed the risk score formula based on the expression of each included gene, weighted by its multivariable Cox regression analysis coefficient, using the following format: Risk scores of the prognostic risk score model (X: coefficient, Y: gene expression level) = 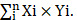
Subsequently, the risk score was computed for every individual. Using the median risk score as a threshold, the training cohort was categorized into a low risk group (below the median risk score) and a high risk group (above the median risk score). The Kaplan-Meier formula in the R package Survival (version 3.3-1) was utilized to generate survival curves, and the log-rank test 54,62-64, was employed to compare the two groups. The model's predictive accuracy was studied using analysis of Receiver Operating Characteristic Curve (ROC) curves 65-67. The independent prognostic value of risk scores and other clinical characteristics were evaluated using Cox regression 68-70. Using the "rms" R package (version 6.7-0), a column chart based on risk scores and clinical characteristics was constructed to provide a reference for predicting the prognosis of patients, and calibration plots were used to assess the chart's prognostic power 71,72.

### Immunoassay of TME

The expression data (ESTIMATE R package (version 1.0.13)) method 73 was used to estimate the stromal score, immune score, ESTIMATE score and tumor purity in HNSCC tissues. Next, the Cell Type Identification for Estimating Relative Subpopulations of RNA Transcripts (CIBERSORT R package (version 0.1.0)) algorithm 74 was used to analyze RNA-Seq data from patients to determine the relative proportion of 22 infiltrating immune cells. In addition, to quantify immune cell infiltration in each sample, the xCell package was used to assess the enrichment of immune cells in HNSCC samples. We then calculated the correlation between risk scores and immunomodulatory genes, particularly immune checkpoints.

### Genetic variant analysis

To identify the copy number variation (CNV) load, we used the InferCNV R package 75 to calculate a large-scale chromosomal CNV score for tumor cells. This analysis entailed assessing relative gene expression in conjunction with chromosomal location data to determine the CNV status of chromosomes within individual cells. This methodology allowed for the effective differentiation of malignant tumor cells from normal cells. To perform this analysis, we prepared raw count matrices, annotation files, and gene/chromosome location files according to the data requirements (https://github.com/broadinstitute/inferCNV). We chose normal cells as a reference and performed an InferCNV analysis using the default parameters to determine whether other malignant cells exhibited substantial chromosomal CNV.

DNA sequencing data was processed by a proprietary bioinformatics platform to identify multiple genomic abnormalities, including single nucleotide variants (SNVs), insertions/deletions, somatic copy number alterations (SCNAs), and translocations. After alignment and deduplication analysis, the Mutect and Vardict SNV tools were used to identify SNVs and insertions/deletions requiring allele score thresholds >0.05 and variant support reads >3. All SNVs and insSertions/deletions were called only within genomic regions to ensure capture accuracy. Thresholds for SCNAs were defined as >2-fold (gain) and 0.5-fold (loss), which were calculated using the CNVkit tool. Translocation variants were then identified by recognizing overlaps of at least four supporting reads, using the NovoBreak and Lumpy tools. Six alternative subtypes were summarized for each sample: C > A, C > G, C > T, T > A, T > C and T > G.

To calculate the TMB, we only considered SNVs that had an allelic fraction of 10% or higher. The TMB was then determined by the number of nonsynonymous SNVs per megabase after applying standard filters. These assays have been validated in previous studies. GENEKEEPER, a proprietary management tool and database, was utilized to oversee and annotate all genetic alterations, ensuring their pathogenicity and clinical significance.

### Drug sensitivity analysis

Using the pRRophetic R package (version 0.5) 76, we utilized the GDSC database (https://www.cancerrxgene.org/), which is the most extensive pharmacogenomics database, to forecast the responsiveness of each tumor sample to treatment. Regression was used to obtain the estimated IC50 values for each drug, and the accuracy of both regression and prediction was tested using 10-fold cross-validation with the GDSC training set. All parameters were selected as default values, including the "combat" to remove batch effects and the mean value of duplicate gene expression.

### Cell culture

The CNE2 cell line and HNE2 cell line were procured from the ATCC. These cellular entities underwent cultivation within F12K medium, enriched with 10% fetal bovine serum sourced from Gibco BRL, USA, and supplemented with 1% streptomycin/penicillin. Notably, the former was nurtured in F12K medium, while the latter found its growth medium in PRMI1640, both from Gibco BRL, USA. The incubation transpired under standardized conditions, maintaining a temperature of 37°C, a carbon dioxide concentration of 5%, and humidity levels at 95%.

### Cell transfection

The reduction of JAG1 expression was accomplished using a small interfering RNA (siRNA) construct acquired from GenePharma in Suzhou, China. The transfection procedure followed the specified protocol for Lipofectamine 3000 RNAiMAX, developed by Invitrogen, USA. Cells were seeded into 6-well plates at 50% confluence and subsequently infected with negative control (si-NC) or knockout constructs (si-JAG1-1 and si-JAG1-2). Each transfection utilized Lipofectamine 3000 RNAiMAX from Invitrogen, USA.

### Cell viability assay

Following transfection, the cellular viability of both CNE2 and HNE2 cells were assessed employing the CCK-8 assay. Cell suspensions were seeded into 96-well plates at a density of 5×10^3^ cells per well and allowed to incubate for a duration of 24 hours. Subsequently, 10 μL of CCK-8 Marker (A311-01, Vazyme) was introduced into each well, followed by an incubation period of 2 hours at 37°C under light protection. To gauge cell viability on days 1, 2, 3, and 4 post-incubations, the absorbance at 450 nm was methodically gauged utilizing an enzymatic marker (A33978, Thermo) 77. The resultant mean optical density (OD) values were computed and depicted graphically as a line chart.

### 5-Ethynyl-2'-deoxyuridine (EDU) proliferation assay

The transfected CNE2 and HNE2 cells were seeded into 6-well plates at a density of 5×10^3^ cells per well and cultured overnight. Subsequently, a 2× EdU working solution was prepared by combining 10 mM EdU with serum-free medium. This solution was added to the cell culture medium, and the cells were allowed to incubate at 37°C for a duration of 2 hours. Following this incubation period, the medium was aspirated, and the cells were gently washed with PBS. Subsequently, the cells were fixed with 4% paraformaldehyde for 30 minutes. A treatment with glycine (2 mg/mL) and 0.5% Triton X-100 for 15 minutes ensued. The cells were then incubated with a mixture of 1 ml 1X Apollo and 1 ml 1X Hoechst 33342 for 30 minutes at room temperature. To quantify cell proliferation, fluorescence microscopy was employed for observation and analysis.

### Wound healing

Upon transfection, the cells were seeded into 6-well plates and cultivated until reaching a cell density of 95%. Subsequently, a sterile 200 μL pipette tip was utilized to meticulously create a linear scratch across the cell layer within the culture wells. Following this, the wells were gently washed with PBS. Post-rinse, the culture medium was replaced, and the cell culture was allowed to proceed. Photographic documentation of the scratches was undertaken at both the 0 hours and 48 hours thereafter. The width of the scratches was measured for subsequent analysis.

### Transwell assay

Prior to the experiment, cells experienced 24 hours of serum deprivation in a serum-free medium. Subsequently, after administering matrix gel from BD Biosciences, USA, the cell suspension was placed in the upper chamber (Costar), while the bottom chamber was supplied with a serum-enriched medium. Subsequent to this arrangement, the cells were incubated for a period of 48 hours. Upon completion of the incubation period, the cells were carefully preserved with a 4% paraformaldehyde solution. Subsequently, a staining procedure was conducted utilizing crystal violet to assess and quantify the invasive properties of the cells.

### Statistical analysis

We performed statistical analysis using R software and Python software to analyze the database data. All *p*-values reported in this study are two-tailed, with values less than 0.05 considered statistically significant. *P*-values below 0.001 were considered highly significant, while those below 0.0001 were regarded as extremely significant.

## Results

### Analysis of cells in NPC

**Figure 1:**
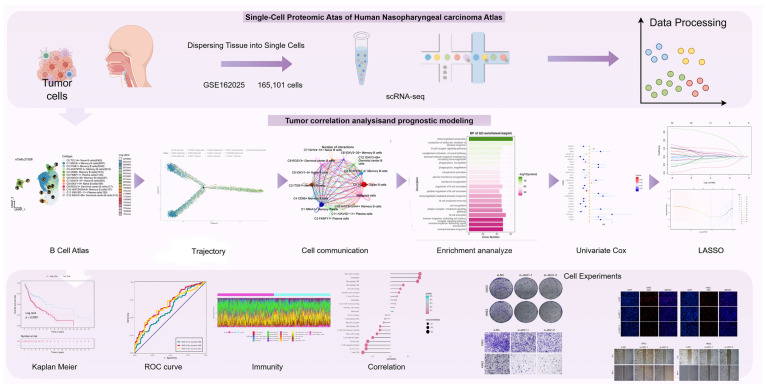
** Graphical Abstract.** The flowchart of this study was shown in the figure.

We did scRNA-seq analysis on tumor tissues and matched peripheral blood mononuclear cells to learn more about how complicated TME is in NPC. After applying rigorous quality control filtering, a total of 165,101 cells were preserved. Furthermore, in order to categorize sets of cells with comparable expression patterns, we conducted an unsupervised cluster analysis using Seurat software. This analysis allowed us to distinguish each cluster into distinct cellular subgroups based on the expression of highly variable genes and characteristic markers. These markers include T/NK cells, B cells, Plasma cells, Myeloid cells, Mast cells, and Malignant cells, which are commonly found in both tumor and peripheral blood samples. The specific flowchart of this study is shown in Figure 1.

### Analysis of B cell subpopulations in NPC

**Figure 2:**
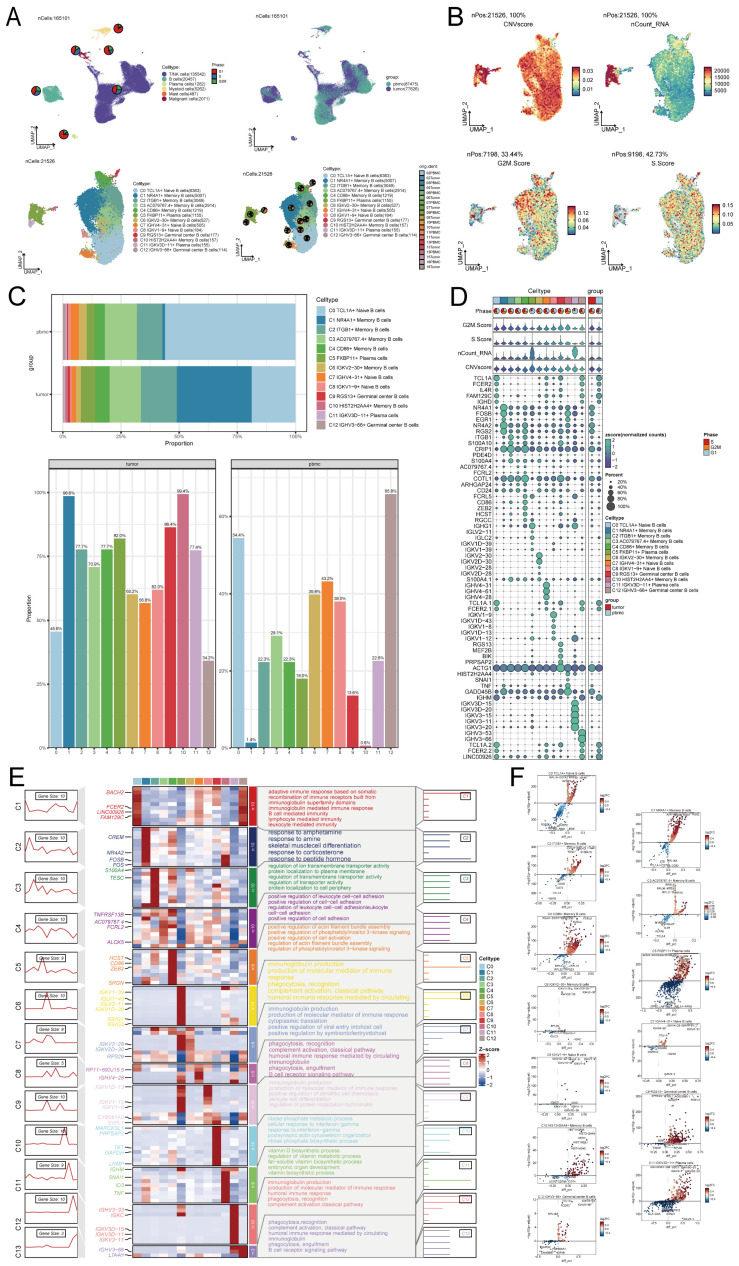
** Single-cell analysis in NPC. (A)** Based on the UMAP plot analyzed, a total of 165,101 individual cells were categorized into six distinct cell types. The pie plots visually depicted the distribution of cell cycle phases (G1, S, and G2/M) for each cell type, showcasing the proportions within each phase (top left). UMAP colored according to cell tissue source (peripheral blood or tumor) (top right). UMAP plot of 21,526 B cells, classifying B cells into 13 major B cell types and pie plots of the sample origin for each cell type (bottom left and bottom right). Each dot represents a cell, colored according to cell types. **(B)** UMAP plots showing CNVscore, ncount_RNA, G2/M.Score, and S.Score for each B cell. Each dot represented a cell, and the color depth from blue to red represents low to high scores. **(C)** Bar graphs showed the proportion of each of the various B cell subpopulations in different sample sources. **(D)** Bubble graph showed the expression of top 5 marker genes of various B cell subpopulations. **(E)** Heatmap showed the differential genes of the 13 cell subpopulations and their corresponding GO-BP enrichment analysis results, yielding the active biological processes in which each B cell respective is located. **(F)** Volcano plots demonstrated the differential genes of each B cell type.

Considering the abundance of B cells in NPC tumor samples and their anti-tumor capacity, we explored the heterogeneity of the entire B cell population. We used cluster analysis to categorize all 21,526 B cells into 13 subpopulations, including C0 TCL1A+ Naive B cells, C1 NR4A1+ Memory B cells, C2 ITGB1+ Memory B cells, C3 AC079767.4+ Memory B cells, C4 CD86+ Memory B cells, C5 FKBP11+ Plasma cells, C6 IGKV2-30+ Memory B cells, C7 IGHV4-31+ Naive B cells, C8 IGKV1-9+ Naive B cells, C9 RGS13+ Germinal center B cells, C10 HIST2H2AA4+ Memory B cells, C11 IGKV3D-11+ Plasma cells and C12 IGHV3-66+ Germinal center B cells (Figure 2A). Differences in CNVscore, nCount-RNA, G2/M.Score and S.Score of B cells were demonstrated using UMAP plots (Figure 2B). The cell ratio bar graph revealed that the C0 TCL1A+ Naive B cells subpopulation accounted for the largest proportion of samples of peripheral blood origin, and the C1 NR4A1+ Memory B cells subpopulation accounted for the largest proportion of samples of tumor origin. Except for the C0 TCL1A+ Naive B cells subpopulation and C12 IGHV3-66+ Germinal center B cells, which accounted for a smaller proportion of tumor-derived samples than peripheral blood-derived samples, all other B cell subpopulations accounted for a greater proportion of tumor-derived samples than peripheral blood-derived samples (Figure 2C). Bubble plots demonstrated the expression of marker genes (top5) for B cell subpopulations (Figure 2D).

### GO-BP enrichment analysis of B cell subpopulations

Enrichment analysis of different genes in the B cell subpopulation revealed that the C0 TCL1A+ Naive B cells exhibited a significant difference in the expression of marker genes, potentially linked to an adaptive immune response involving the rearrangement of immune receptors composed of immunoglobulin superfamily domains. This response also involves immunoglobulin-mediated immune response, B cell-mediated immunity, lymphocyte-mediated immunity, and leukocyte-mediated immunity. Additionally, the C4 CD86+ Memory B cells subpopulation may be associated with the positive regulation of actin filament bundle assembly, phosphatidylinositol 3-kinase signaling, cell activation, as well as the regulation of actin filament bundle assembly and phosphatidylinositol 3-kinase signaling. These biological processes are depicted in Figure 2E. The volcano plot demonstrates the differential expression of marker genes in B cell subpopulations (Figure 2F).

### GSEA enrichment analysis of B cell subpopulations

**Figure 3:**
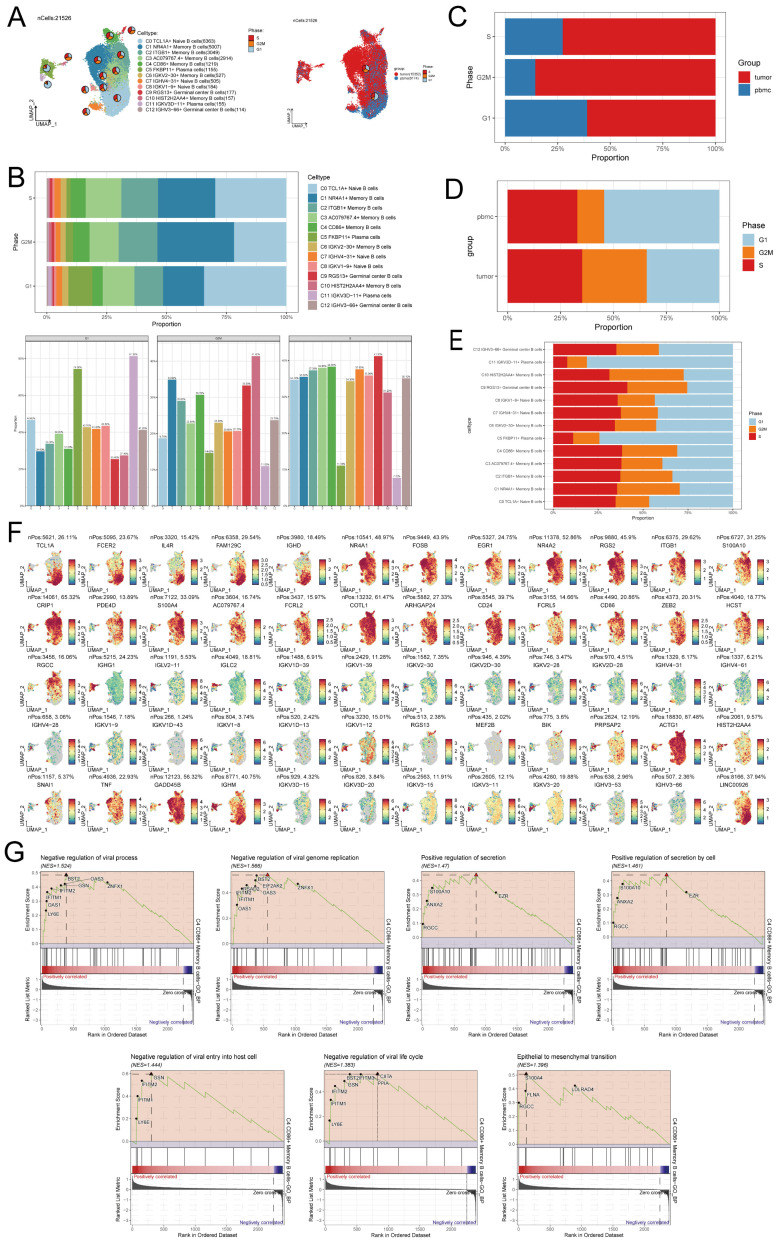
** GSEA enrichment analysis of B cell subpopulations. (A)** UMAP plots of 21,526 B cells, classifying B cells into 13 major B cell types and coloring them according to their cell types and origin from peripheral blood or tumor. The pie plots visually depicted the distribution of cell cycle phases (G1, S, and G2/M) for each cell type and sample sources. **(B)** Bar graphs showed the proportion of various B cell subpopulations in each of the different cell phases. **(C)** Bar graph showed the proportion of B cells from different cell phases in different sample. **(D-E)** Bar graph showed the proportion of B cells of different cell phases origin in different samples (D) and cell types (E). **(F)**UMAP plots demonstrated the expression of marker genes in the subpopulations of B cells. **(G)** GSEA analyzed of C4 CD86+ Memory B cells.

Additional investigation into the B cell subpopulations indicated that most B cells originated from tumor samples, with only a minority originating from peripheral blood samples. Among the B cells derived from peripheral blood samples, the predominant subpopulation was C0 TCL1A+ Naive B cells, while the remaining B cell subpopulations were primarily derived from tumor samples (Figure 3A).

It can be seen from the bar chart that the proportion of C5 FKBP11+ Plasma cells subpopulations in G1 phase is higher than that in other cell cycles, and the proportion of C4 CD86+ Memory B cells subpopulations in G1 phase is lower than that in other cell cycles. However, such a result may be affected by the total number of cells. We observed the proportion of different cell cycles in different B cell subpopulations, and found that the G1 phase (74.30%) of C5 FKBP11+ Plasma cells subpopulations was indeed higher than the G2/M phase (14.60%) and S phase (11.10%). The S phase (38.00%) of C4 CD86+ Memory B cells was higher than that of G1 phase (31.00%) and G2/M phase (30.70%)(Figure 3B).The proportion of B cells of each cell cycle was higher in the tumor sample-derived B cells than in the peripheral blood sample-derived ones, which might be the reason why the number of tumor-derived B cells was significantly more than the number of peripheral blood sample-derived B cells (Figure 3C).The bar graph of the proportions of B cells with different cell cycles in the different sample sources showed that the proportion of B cells in G2/M phase was higher in the tumor-source than in the peripheral blood-source, while the proportion of B cells in G1 phase was lower in the tumor-source than in the peripheral blood-source (Figure 3D). Among the cell cycle ratios of all B cell subpopulations, it was clear that the cell S phase of C5 FKBP11+ Plasma cells and C11 IGKV3D-11+ Plasma cells subpopulations was shorter than average (Figure 3E). UMAP plots demonstrated the expression of top5 marker genes of each B cell subpopulation (Figure 3F). The GSEA analysis examined the distinct genes of the C4 CD86+ Memory B cells subpopulation and revealed their significant involvement in inhibiting viral processes, controlling viral genome replication, promoting secretion, enhancing secretion by cells, and preventing viral entry into host cells. Figure 3G indicates that C4 CD86+ Memory B cells have the potential to hinder viral invasion and activity by negatively regulating the viral life cycle and Epithelial to mesenchymal transition, two of the seven biological pathways that exhibited noteworthy enrichment outcomes.

### Monocle trajectory analysis of B cell subpopulations

**Figure 4:**
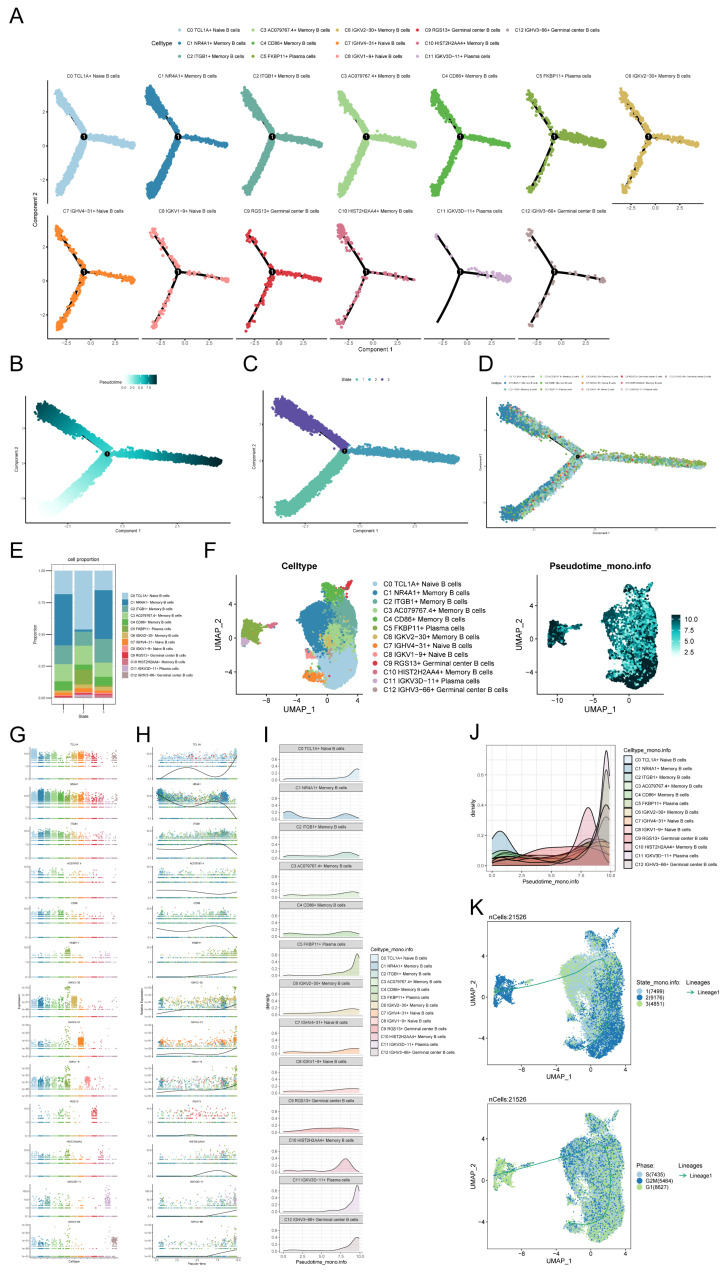
** Trajectory analysis of B cell subclusters. (A)** The temporal trajectory of 13 B-cell subclusters was inferred, with each point representing a cell colored according to its cluster label. The developmental trajectories of different cell subpopulations are shown. **(B)** Plot of the proposed temporal trajectory analyzed of the B cell subpopulations. Each point represented an individual cell, showed the pseudo-temporal scores of each cell from white to dark green, indicated the early and late states, respectively. **(C)** Monocle inferred that there were three states in the process of B cell development. **(D)** The distribution of B cell subpopulations in the developmental trajectory inferred by Monocle.** (E)** Bar graph showed the proportion of each of the different B cell clusters in each of the different states, respectively. **(F)** UMAP plots showed the pseudotemporal scores of each B cell from white to dark green, indicated early and late states, respectively. **(G)** Expression of marker genes in the 13 B cell subpopulations. **(H)** Changed in pseudotemporal scores of each marker gene. **(I-J)** A ridge diagram subplot of the number of cells of the 13 B cell subpopulations identified throughout pseudotemporal time using Monocle. **(K)** The differentiation state of B cellsubpopulations was inferred by Slingshot (top), and the distribution of differentiation trajectory in different phases of B cells was plotted (bottom). Solid lines represented the trajectory of differentiation, and arrowed represent the direction of differentiation.

To depict the temporal dynamics of B cells during NPC formation, individual cells were reordered into a pseudotemporal timeline using the Monocle toolkit, which clearly showed that C0 TCL1A+ Naive B cells, C1 NR4A1+ Memory B cells, C2 ITGB1+ Memory B cells, C3 AC079767.4+ Memory B cells and C4 CD86+ Memory B cells subpopulations developed uniformly on the pseudotimeline, while other B cell subpopulations had different trends on the pseudotimeline (Figure 4A). We observed that most cells at the beginning of the pseudotimeline were State1 by derivation, with State2 and State3 appearing along the progression of the proposed timeline (Figures 4B-D). We probed the proportion of each of the different B cell clusters in the different states, respectively, and found that State2 had a much smaller proportion of C1 NR4A1+ Memory B cells than State1 and State3, while State2 had a much larger proportion of C0 TCL1A+ Naive B cells than State1 and State3 (Figure 4E). The UMAP plot demonstrated the proposed temporal status of all B cells (Figure 4F). In order to investigate the variations in the quantity of each B cell subpopulation over time, we initially examined the presence of marker genes within the B cell subpopulations (Figure 4G). Subsequently, we analyzed the expression of distinct marker genes throughout the suggested temporal alterations (Figure 4H). As we saw previously in the figure, in the early stage of NPC development, most cells were C1 NR4A1+ Memory B cells, while in the later stage of development, there appeared mostly C11 IGKV3D-11+ Plasma cells and C12 IGHV3-66+ Germinal center B cells, etc. (Figures 4I, J). The inferred cellular development trajectories, as determined by Slingshot, exhibited a general concordance with the findings from Monocle (Figure 4F, K). Notably, during the early stages of differentiation, B cells in the G1 phase predominated, while throughout the course of disease progression, there was a gradual augmentation in the population of B cells in the S phase and G2/M phase.

### Intercellular communication between malignant cells and B cell subpopulations

**Figure 5:**
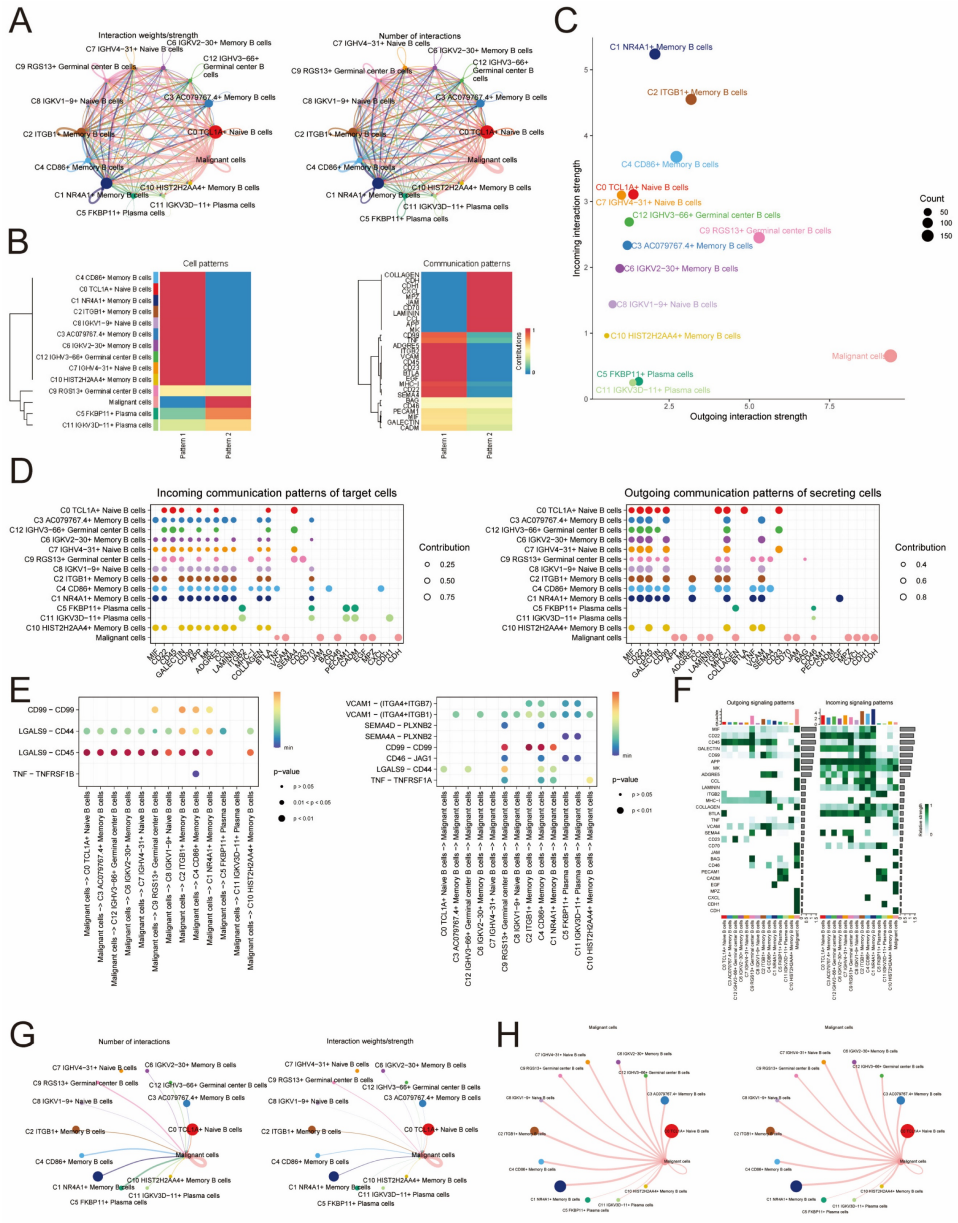
** Intercellular communication interactions between malignant cells and B cell populations. (A)** Plot of net counts of interactions between B cell subpopulations and malignant cells versus interaction weights. The thicker the indicated line, the higher the number of interactions and the stronger the weight/strength of the interaction between the two cell types. **(B)**Heatmaps of cellular communication patterns between B cell subpopulations and malignant cells, showed the correspondence between inferred potential patterns and cell populations, as well as signaling pathways. Shades of color indicated the contribution of cell populations or signaling pathways to each potential pattern. **(C)** The scatter plot depicted the communication network analyzed between B cells and malignant cells, the color of the dots indicated different cells and the size of the dots indicated the number of cells. **(D)** Bubble plots showed afferent versus efferent communication patterns between B cell subpopulations and malignant cells. **(E)** Bubble plots demonstrated significant ligand-receptor pairs between malignant cells and different B cells. Dot color indicates the communication probability and dot size indicates the calculated p-value. Blank means that the communication probability is zero. *p*-value is calculated based on the one-sided substitution test. **(F)** Heatmaps demonstrated the strength of signaling interactions between B cells and malignant cells in the cellular communication network. **(G)** Circle diagram of B cell subpopulations acted on the malignant cell communication network. The thicker the indicated line, the higher the number of interactions and the stronger the weight/strength of the interaction between the two cell types.** (H)** Malignant cell acted on B cell subpopulation communication network circle diagram. The thicker the line indicated, the greater the number of interactions, and the stronger the interaction weight/intensity between the two cell types.

Based on the net counts of interaction plots and interaction weight plots of B cell subpopulations with malignant cells, it was found that both in terms of net counts of interactions and interaction weights/strengths, malignant cells exhibited stronger interactions with individual B cell subpopulations compared to interactions between individual B cell subpopulations (Figure 5A).

Specifically, cellular communication signaling in malignant cells, C5 FKBP11+ Plasma cells and C11 IGKV3D-11+ Plasma cells subpopulations was mainly driven by pattern2, which includes signaling pathways such as COLLAGEN, CDH and CXCL. In contrast, cell communication signaling in other B cell subpopulations was mainly driven by pattern1, which included signaling pathways such as CD99, SEMA4 and VCAM (Figure 5B). In Figure 5C, it was observed that the malignant cell-associated pathway had a considerably higher Outgoing interaction strength compared to B cells, while its Incoming interaction strength was lower than most B cells. According to the CellChat analysis, various cells can function as primary communication centers in various signaling pathways. In this research, cancerous cells were identified as the main hubs for cellular interactions, receiving numerous incoming signals like VCAM, JAM, CD46, EGF, MPZ, and CDH, and transmitting outgoing signals such as MK, APP, and CCL. Cell-cell interaction centers with afferent signals such as MK, APP and CCL. In contrast, B cells displayed intercellular interaction centers with numerous incoming signals like MIF and CD22, along with outgoing signals like CD99 (Figure 5D). CellChat identified several important ligand-receptor pairs such as CD99-CD99, LGALS9-CD44, LGALS9-CD45, and TNF-TNFRSF1B, which are the most important ligand-receptor pairs for the interaction of malignant cells acting on B cells are the most important signaling pathways. On the other hand, ligand-receptor pairs such as VCAM1-(ITGA4+ITGB7), TNF-TNFRSF1A, LGALS9-CD44, CD46-JAG1, CD99-CD99, SEMA4A-PLXNB2, SEMA4D-PLXNB2, and VCAM1-(ITGA4+ITGB1) were determined to be the B cells acting on malignant cells as the most important signaling pathways. This information contributes to the understanding of the communication process from malignant cells to B cells and from B cells to malignant cells (Figure 5E). In general, the signaling pathway of cancerous cells exhibited greater strength in the efferent signaling pattern aspect, while the B cell signaling pathway exhibited greater strength in the afferent signaling pattern (Figure 5F). With the exception of C7 IGHV4-31+ Naive B cells, cells of all B cell subpopulations could act on malignant cells (Figure 5G). And malignant cells could act on all B cell subpopulations (Figure 5H).

### Signaling pathways CD99 and SEMA4 in cellular communication

**Figure 6:**
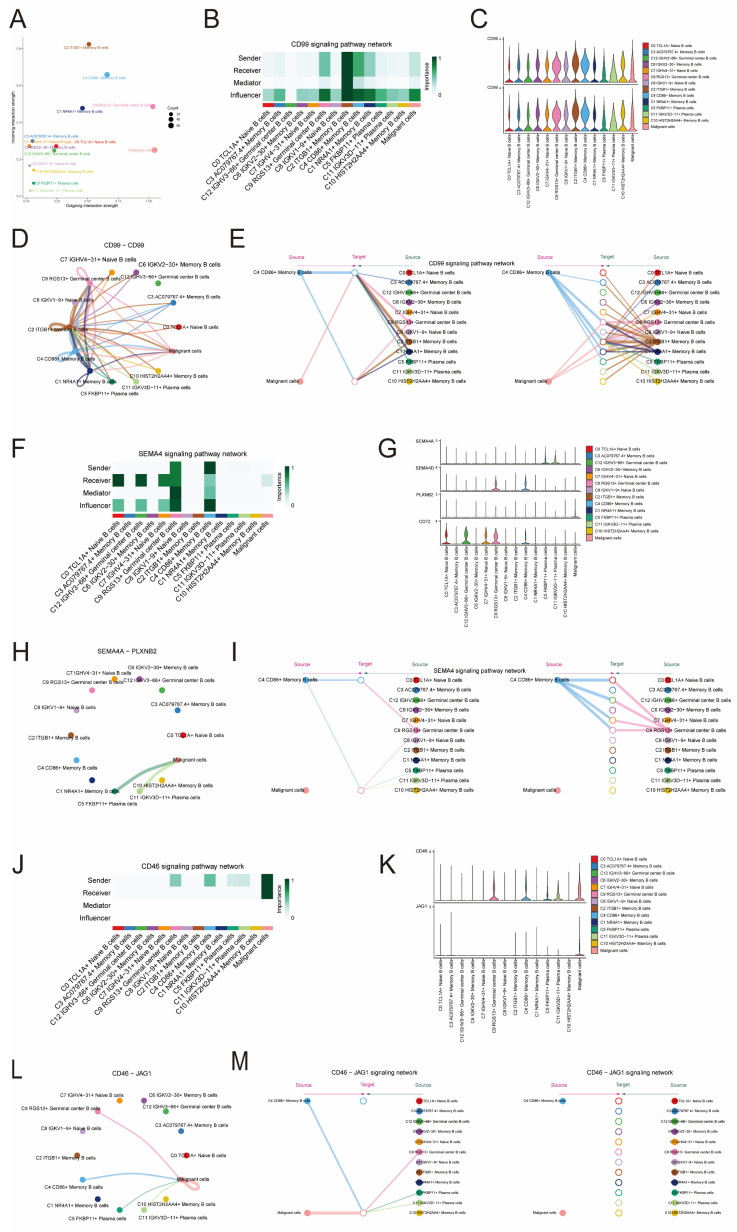
** Signaling pathways Role of CD99 and SEMA4 in cellular communication. (A)**The signaling pathways associated with CD99 and SEMA4 were projected onto a two-dimensional manifold based on cellular subpopulations. The color of the dots indicated different cells and the size of the dots indicated the number of cells. **(B)** Heatmap showed the relative importance of each cell group based on the computed four network centrality measures of CD99 signaling network. **(C)** Violin plot showed the expressed role of the signaling pathway CD99 in B cell and malignant cell communication. **(D)** Network circle diagram of the role of the signaling pathway CD99 in B cell and malignant cell communication. **(E)**Hierarchical plot showed the inter-cellular communication network between malignant and B cells inferred through a typical CD99 signaling pathway. **(F)** Heatmap showed the relative importance of each cell group based on the computed four network centrality measures of SEMA4 signaling network. **(G)** Violin diagram showed the role of the signaling pathway SEMA4 in B cell and malignant cell communication processes. **(H)** Network circle diagram played the role of the signaling pathway SEMA4 in B cell and malignant cell communication processes. **(I)** Hierarchical diagram showed the inter-cellular communication network between malignant and B cells inferred through typical SEMA4 signaling pathways. **(J)** The heatmap displayed the centrality scores of the CD46 signaling pathway.** (K)** The violin plot illustrated the interactions between ligand-receptor pairs.** (L)** The circular diagram presented the intercellular communication network of the CD46-JAG1 ligand-receptor pair with macrophages as the recipients.** (M)** The hierarchical diagram depicted the communication panel network among various cell types within the CD46-JAG1 pathway.

To explore in depth, the detailed communication functions of individual signaling pathways and the coordinated functions between multiple cell populations and signaling pathways, we used CellChat to determine the roles that the three cellular pathways, CD99, SEMA4, and CD46, play in signaling between different cells. In Figure 6A, it is evident that the signaling pathway of cancerous cells exhibits limited senders and numerous receiver pathways, whereas the signaling pathway of C4 CD86+ Memory B cells displays abundant senders and a limited number of receiver pathways. Furthermore, our findings indicated that within the CD99 signaling pathway, malignant cells functioned as influencers in intercellular communication, while C4 CD86+ Memory B cells served as influencers, senders, and receivers in this communication process (Figure 6B). Notably, CD99 expression was present in all B cell subpopulations and malignant cells (Figure 6C). Using CellChat analysis, it was found that malignant cells could interact with C9 RGS13+ Germinal center B cells, C8 IGKV1-9+ Naive B cells, C2 ITGB1+ Memory B cells, C4 CD86+ Memory B cells and C1 NR4A1+ Memory B cells. These five B cells communicated with each other through the CD99-CD99 signaling pathway, which did not exist with other B cells (Figure 6D). By analyzing the centrality of the CD99 signaling pathway in the network, we discovered that C4 CD86+ Memory B cells and malignant cell populations have the ability to facilitate communication among B cells and facilitate communication from malignant cells to B cells. The complexity and redundancy of the CD99 signaling network in NPC is indicated, as there are various cell populations originating from ligands that can target B cells of NPC (Figure 6E). Conversely, within the SEMA4 signaling pathway, cancerous cells can function as recipients of intercellular communication, while C4 CD86+ Memory B cells primarily serve as influencers and transmitters of intercellular communication, in addition to their roles as mediators and recipients (Figure 6F). Notably, although SEMA4 was expressed in all these cells, the lower expression levels may imply that it has a relatively weak role in signaling (Figure 6G).

Through CellChat analysis, it was discovered that solely C11IGKV3D-11+ Plasma cells and C5 FKBP11+ Plasma cells had the ability to engage in one-way communication with malignant cells via the SEMA4A - PLXNB2 signaling pathway. In contrast, the remaining B cell populations were incapable of communicating with malignant cells through this specific signaling pathway (Figure 6H). Through network centrality analysis, it was discovered that C4 CD86+ Memory B cells have the ability to facilitate communication among B cells. However, there was no evidence of any communication process between malignant cells (ligand) and B cells (receptor). This suggested that the SEMA4 signaling network in NPC was relatively simple and does not possess the high degree of redundancy of the aforementioned CD99 signaling pathway (Figure 6I). Within the CD46 signaling pathway, cancerous cells have the ability to function as both transmitters and recipients of intercellular communication, while C4 CD86+ Memory B cells primarily function as transmitters (as shown in Figure 6J). Notably, although CD46 was expressed in all these cells, the lower expression level may imply its relatively weak role in signaling. JAG1 was only expressed in individual cell subpopulations and at low levels (Figure 6K). Through the analysis of CellChat, it was discovered that JAG1 could be expressed by only specific B cell types, namely C4 CD86+ Memory B cells, C5 FKBP11+ Plasma cells, C9 RGS13+ Germinal center B cells, and C11 IGKV3D-11+ Plasma cells, using the CD46-JAG1 signaling pathway for one-way communication with cancerous cells. However, other B cell subpopulations were unable to establish communication with malignant cells through this pathway (Figure 6L). Analysis of the CD46 signaling network's network centrality indicated that the communication process from malignant cells (ligand) to B cells (receptor) could be driven by C4 CD86+ Memory B cells and malignant cells, while no communication process existed among B cells (Figure 6M).

### Marker genes of C4 CD86+ memory B cells

**Figure 7:**
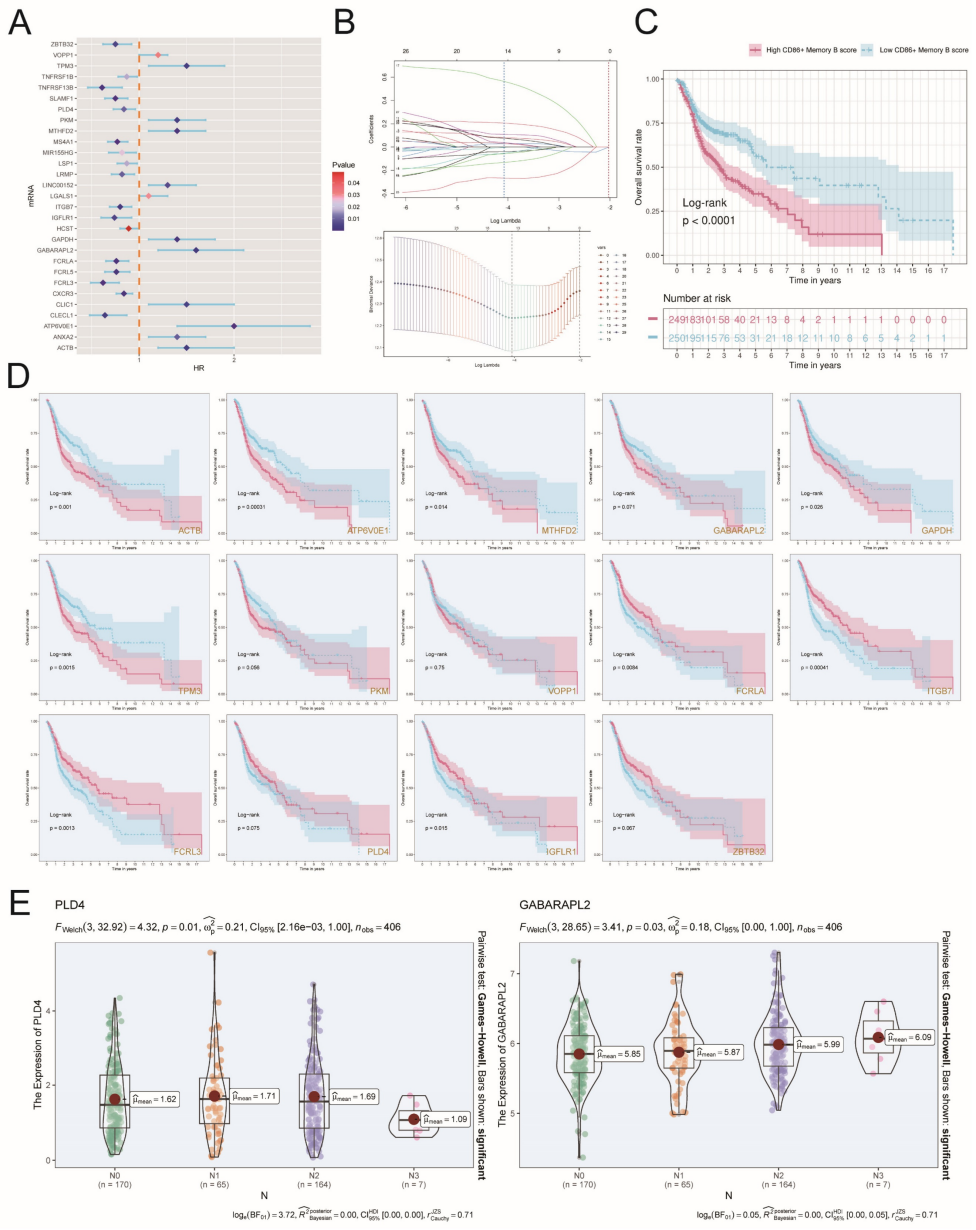
** Construction of a prognostic model associated with C4 CD86+ Memory B cells. (A)** Forest plot of univariate cox regression analysis. **(B)** Through LASSO regression analysis, genes associated with prognosis were selected. The optimal parameter (lambda) was determined through ten-fold cross-validation, and the LASSO coefficient curve was determined by the optimal lambda. **(C)** OS curves for different scoring subgroups. **(D)** OS curves of risk genes. **(E)** Expression of the gene *PLD4* versus the gene *GABARAPL2* in different lymph node metastasis scenarios.

Since we mainly focused on the subpopulation of C4 CD86+ Memory B cells, we selected the top100 differential genes of C4 CD86+ Memory B cells. For better clinical integration, we next identified 29 genes that could be used as prognostic features using univariate Cox regression analysis (Figure 7A). In order to tackle the potential multicollinearity problem among these genes, we utilized a LASSO regression analysis to select 14 out of the 29 DEGs for subsequent analysis (Figure 7B). Afterwards, employing multivariate Cox regression analysis, we developed a score linked to these genes, referred to as the 'CD86+ Memory B score'. Subsequently, we endeavored to forecast the patients' prognosis using this score, aiming to further ascertain its significance. The patients were categorized into a group with a high CD86+ Memory B score and a group with a low CD86+ Memory B score. Surprisingly, we discovered that the group with lower scores had a superior advantage in terms of survival when compared to the group with higher scores (as shown in Figure 7C). Additional investigation into certain genes linked to the prognostic outlook of patients with unveiled that the genes *ACTB, ATP6VOE1, MTHFD2, GAPDH,* and* TPM3* were identified as risk elements, while the genes *FCRLA, ITGB7, FCRL3*, and *IGFLR1* were recognized as protective elements (Figure 7D). In addition, the expression levels of genes varied among patients with different TNM stages. For example, the gene *PLD4* was expressed in stage N1 > stage N2 > stage N0 > stage N3, while the gene *GABARAPL2* was expressed in stage N3 > stage N2 > stage N1 > stage N0 (Figure 7E).

### Construction of risk scores

**Figure 8:**
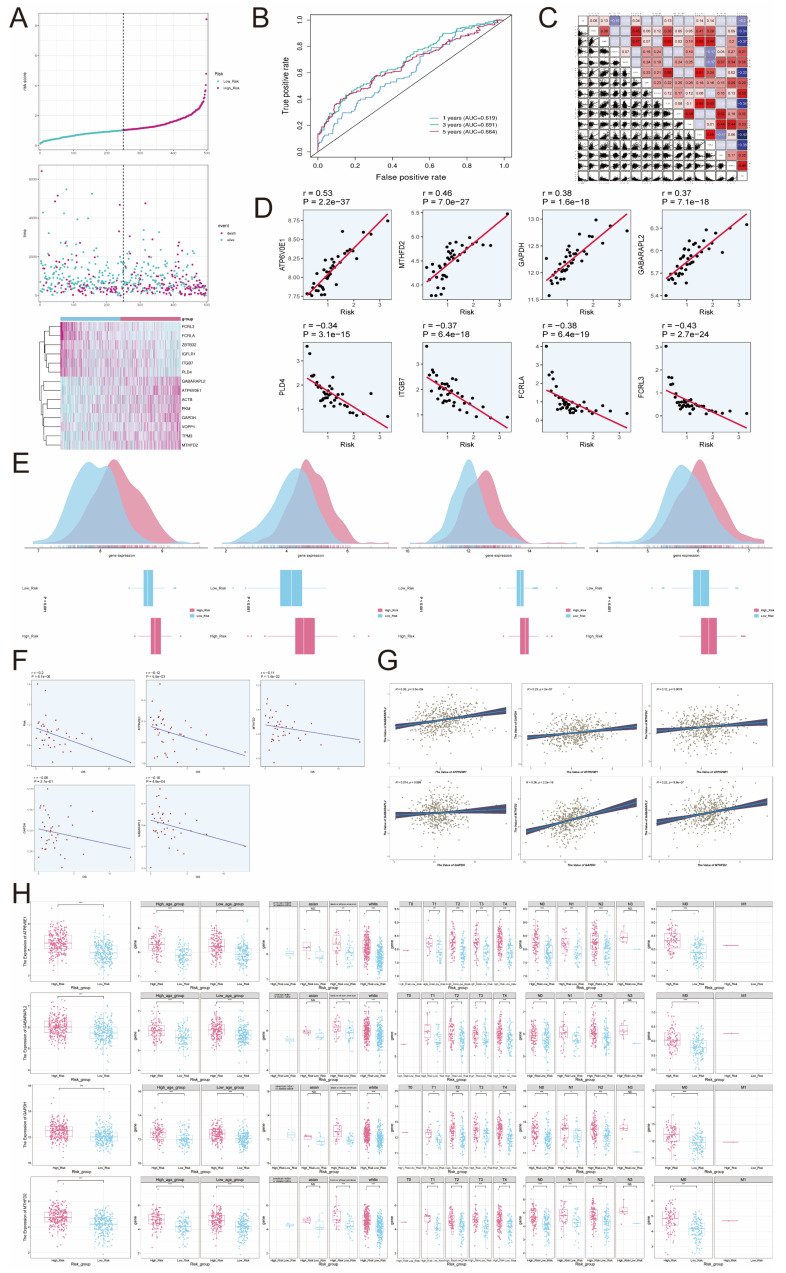
** Construction of Risk Score Model for C4 CD86+ Memory B cells. (A)** Risk profiles of patients. **(B)** ROC curves depicted the sensitivity and specificity of risk scores in predicting 1-, 3-, and 5-year survival in the cohort. **(C)** Overall plot of correlation of individual genes with risk scores. **(D)** Eight genes that showed a significant correlation with the risk scores (*ATP6V0E1, MTHFD2, GAPDH, GABARAPL2, PLD4, ITGB7, FCRLA, FCRL3*). **(E)** Gene expression of these four genes (*ATP6V0E1, MTHFD2, GAPDH, GABARAPL2*) was higher in the high-risk group as compared to the low-risk group. **(F)** Correlation of OS with these four genes and risk score.** (G)** In this cohort, the risk genes were significantly correlated with the risk genes (*ATP6V0E1, MTHFD2, GAPDH, GABARAPL2*) correlation. **(H)** Differential expression of risk genes (*ATP6V0E1, MTHFD2, GAPDH, GABARAPL2*) in the high- and low-risk groups, the high- and low-age groups, the different races groups, and the different TNM stages. *P < 0.05, **P < 0.01, ***P < 0.001, ****P < 0.0001. "ns" was used to say that there was no significant difference.

Based on the aforementioned findings, we examined 14 genes linked to prognosis. Utilizing these genes, we developed risk score characteristics and divided patients into high-risk and low-risk categories, employing the median as the threshold. The expression levels of genes *FCRL3, FCRLA, ZBTB32, IGFLR1, ITGB7*, and* PLD4* were lower in the high-risk group compared to the low-risk group. On the other hand, the expression of genes *GABARAPL2, ATP6VOE1, ACTB, PKM, GAPDH, VOPP1, TPM3,* and *MTHFD2* were higher, which aligned with the previous findings (Figure 8A). To evaluate the sensitivity and specificity of risk scores in predicting the survival of patients for 1, 3, and 5 years, we conducted ROC curve analysis in our group and discovered that the risk scores demonstrated higher accuracy in predicting the mentioned survival subcategories. Specifically, the AUC for 1 year was 0.619, for 3 years was 0.691, and for 5 years was 0.664 (Figure 8B).

To further investigate the impact of different genes on prognosis, we analyzed the relationship between numerous genes and risk scores (Figure 8C). Among them, *ATP6VOE1, MTHFD2, GAPDH,* and *GABARAPL2* showed significant positive correlations with risk scores, with correlation coefficients of 0.53, 0.46, 0.38, and 0.37, respectively. On the other hand, *PLD4, ITGB7, FCRLA,* and *FCRL3* exhibited significant negative correlations with risk scores, with correlation coefficients of -0.34, -0.37, -0.38, and -0.43, respectively (Figure 8D). In Figure 8E, the high-risk group exhibited elevated expression levels of *ATP6V0E1, MTHFD2, GAPDH,* and *GABARAPL2* genes compared to the low-risk group. The risk score was negatively correlated with overall survival (OS), and the expression of these four genes (*ATP6V0E1, MTHFD2, GAPDH, GABARAPL2*) was also negatively correlated with OS (Figure 8F), which suggested that the high expression of these risk genes might be detrimental to the clinical survival of patients. To verify whether these risk genes might interact with each other to cause errors, we investigated the two-by-two correlation between them and found that the two-by-two correlation between these risk genes was not strong (Figure 8G), which indicated that the genes *ATP6V0E1, MTHFD2, GAPDH* and* GABARAPL2* were risk genes with more independent effects on the prognosis of patients. Afterward, we extensively investigated the gene expression of *ATP6V0E1, MTHFD2, GAPDH,* and *GABARAPL2* in the high-risk group, low-risk group, high-age group, low-age group, different ethnicity group, and different TNM stage group. Our findings revealed that the expression levels of *ATP6V0E1, MTHFD2, GAPDH,* and* GABARAPL2* were comparatively increased in the high-risk group compared to the low-risk group (Figure 8H).

### Nomogram construction and validation

**Figure 9:**
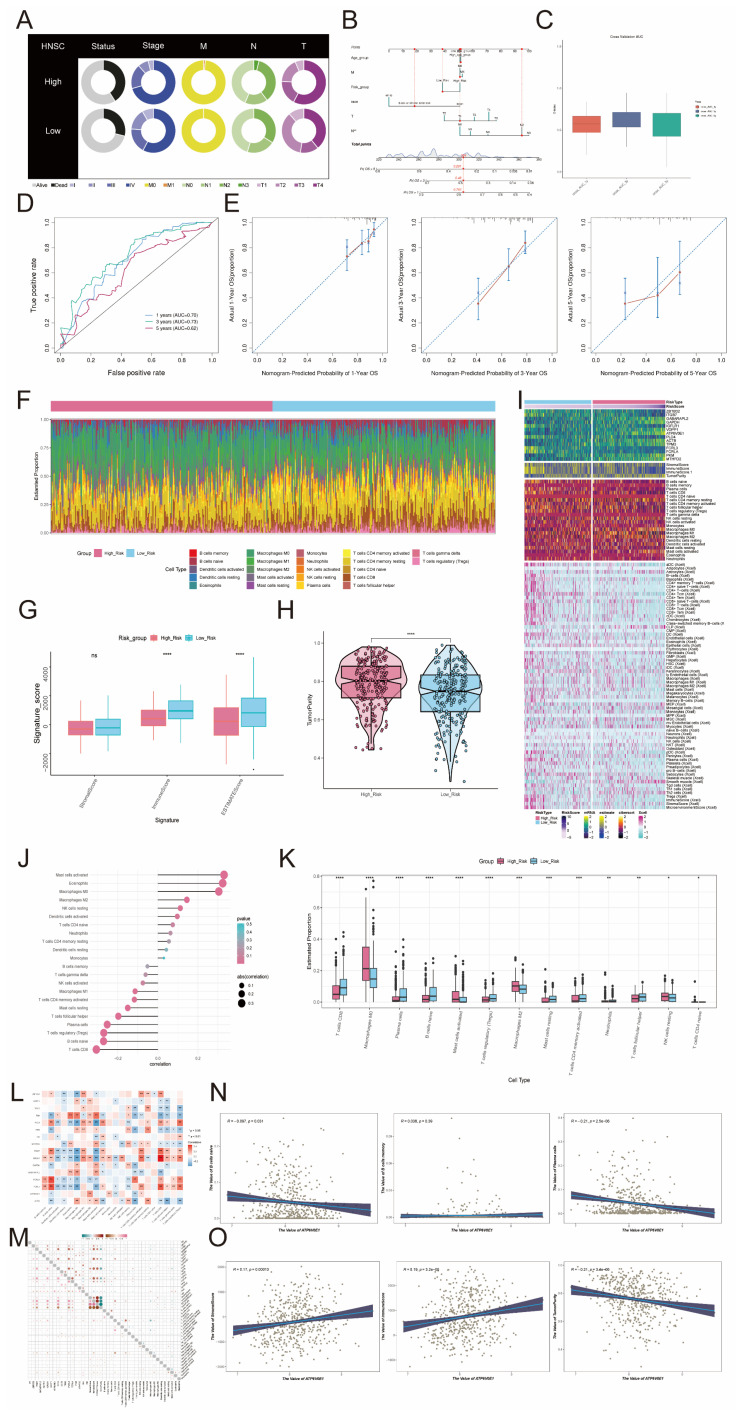
** Survival analysis and TME analysis. (A)** Pie plots of different status, different stages and different TNM stages in high and low risk groups. **(B)** Nomogram showed the prediction of 1, 3, and 5-year of OS based on race, tumor clinical stage (T, M, and N), age, and risk score. **(C)** Box-and-line plots depicted the C-index of the AUC values of the risk scores for predicting 1-, 3-, and 5-year survival. **(D)** ROC curves depicted the sensitivity and specificity of the risk scores for predicting 1-, 3-, and 5-year survival. **(E)** Calibration curves for column charts predicted 1-, 3-, and 5-year OS. The OS predicted by the line plot model was plotted on the x-axis and the actual OS was plotted on the y-axis. **(F)** Proportion of each infiltrating immune cell type in the high- and low-risk groups was shown using CIBERSOFT. **(G)**Stromal score, immune score, and estimate score were calculated for the high- and low-risk groups, respectively, using ESTIMATE. **(H)** TumorPurity was calculated using ESTIMATE for the high and low risk groups, respectively. **(I)** The difference in modeling genes, StromalScore, ImmuneScore, ESTIMATScore, TumorPurity, and the level of immune cell infiltration between the high- and low-risk groups. **(J)**Lollipop chart showed the correlation of immune cell versus risk score. **(K)** The proportions of each infiltrating immune cell type in the high and low risk groups were demonstrated using CIBERSOFT. **(L-M)** The correlation between the risk genes and the 22 immune cells. **(N)** Correlation of the gene *ATP6V0E1* with naive B cells, memory B cells, and plasma cells. **(O)** Correlation of the gene *ATP6V0E1* with Stromal Score, Immune Score, and TumorPurity. *P < 0.05, **P < 0.01, ***P < 0.001, ****P < 0.0001. "ns" was used to say that there was no significant difference.

In Figure 9A, the high-risk group exhibited a greater percentage of patients with mortality status, Stage IV, M1, N3, and T4 in comparison to the low-risk group. Subsequently, we created a Nomogram survival forecast model for the OS of individuals with HNSCC using autonomous prognostic factors to anticipate the prognosis of patients with HNSCC (Figure 9B). The model underwent validation and demonstrated good performance in predicting the OS C index (Figure 9C). To evaluate the accuracy of risk scores in predicting survival at 1-, 3-, and 5-year intervals in patients, we conducted ROC curve analyses in the three cohorts. The results showed a high predictive accuracy, with AUC (1 year) = 0.70, AUC (3 years) = 0.73, and AUC (5 years) = 0.62 (Figure 9D). Additionally, calibration curves were plotted to demonstrate the agreement between predicted and observed values for 1-year and 3-year OS in both the training and validation cohorts (Figure 9E), which showed good agreement.

### Tumor microenvironment analysis

The analysis of CIBERSORT and xCell revealed the immune infiltration in the tumor samples. With the stacked bar diagram, we could observe a difference in the Estimated Proportion of immune cells between the high-risk and low-risk groups (Figure 9F). Using ESTIMATE to calculate the stromal score, immune score and estimate score of the high-risk and low-risk groups, it was found that the immune score and estimate score of the low-risk group were significantly higher than those of the high-risk group, which indicated that the level of immune cell infiltration in tumor samples of the low-risk group was higher, and this might be correlate with its good prognosis (Figure 9G). In addition, TumorPurity was lower in the low-risk group than in the high-risk group (Figure 9H). The heatmap showed that the infiltration levels of multiple immune cells in tumor samples in the low-risk group were significantly higher than those in the high-risk group (Figure 9I). B cells naive, B cells memorys and plasma cells which were immune cells were negatively correlated with risk score (Figure 9J). T cells CD8, Plasma cells, B cell naive, Tregs, Mast cells resting, T cells CD4 memory activated and neutrophils had lower Estimated Proportion in the high-risk group than in the low-risk group, while immune cells such as Macrophages M0 and Macrophages M2 had higher Estimated Proportion in the high-risk group than in the low risk group (Figure 9K).By looking at the heatmap of the correlation between the risk genes and the infiltration of 22 immune cells, we found that the expression of many genes correlated with the infiltration of immune cells (Figure 9L). In order to explore whether genes affect disease prognosis and B cells are related, we studied the correlation between genes and B cells (Figure 9M). Due to the close association between the gene *ATP6V0E1* and prognosis, we investigated the correlation of this gene with naive B cells, memory B cells, plasma cells, as well as its correlation with StromalScore, ImmuneScore, and tumor purity. (Figure 9N-O).

### Identification of DEGs and their enrichment analysis results

**Figure 10:**
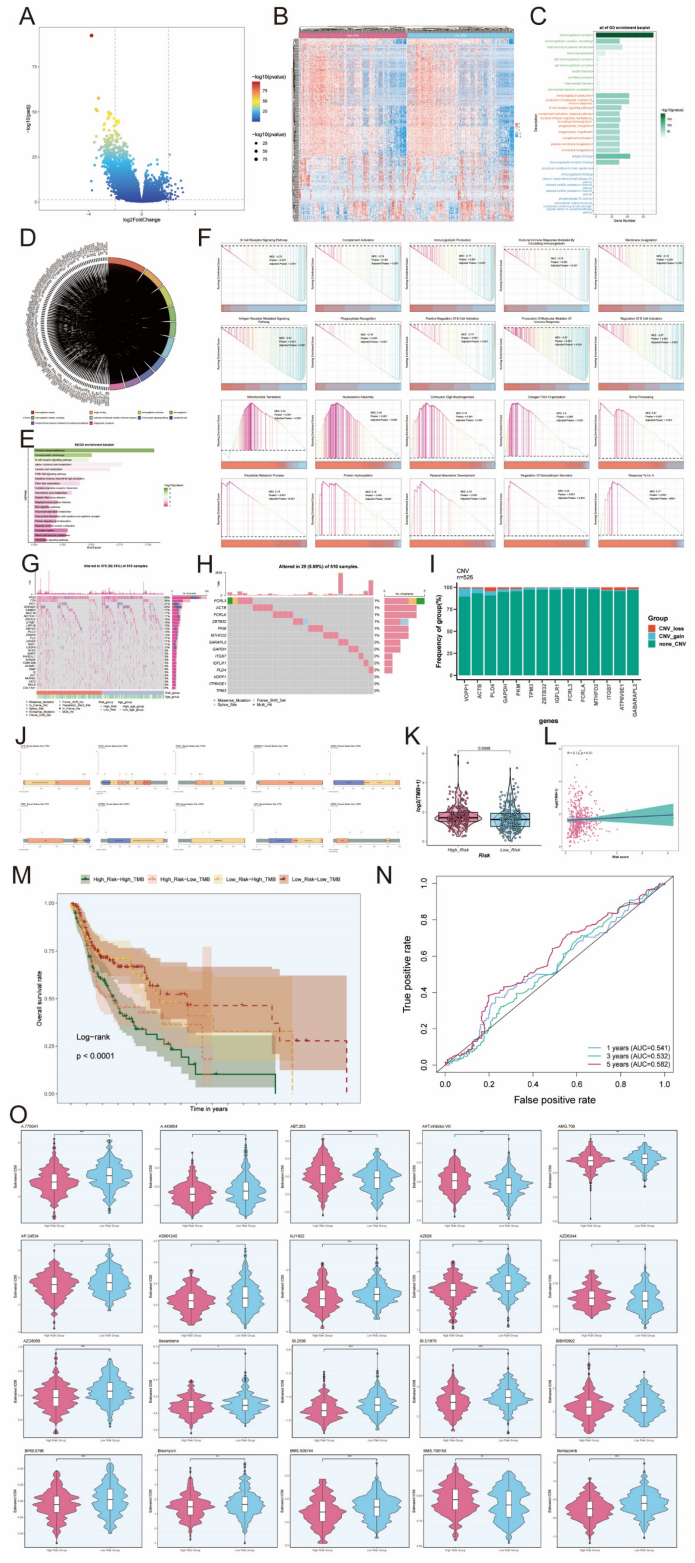
** Identification of DEGs and the results of enrichment analysis. (A)** Volcano plot showed significantly DEGs. Each dot represents a gene. **(B)** Heatmap of DEGs. Each column in the heatmap represented a sample, and each row represented the expression level of a gene. The color scale next to the heatmap indicated from blue (low expression) to red (high expression). **(C)** Bar graph of GO enrichment analysis of DEGs. **(D)** String graph of GO enrichment analysis. It showed the relationship between DEGs and the top 10 enriched GO pathways. **(E)** Graph of the results of KEGG enrichment analysis of differentially expressed pathway. **(F)** Results of GSEA enrichment analysis of DEGs. **(G)** Waterfall plot showing the mutation status of the top 30 high mutation frequency genes in the samples. The top bar graph shows the mutation load of the samples, and the histogram on the right side showed the mutation status of the genes for each mutation type. **(H)** Waterfall plot showed the mutation profile of 14 risk genes in the sample, the upper bar graph showed the mutation load of the genes, and the histogram on the right side showed the mutation type of each of the genes. **(I)** Bar graph showed the CNVs of the 14 risk genes. **(J)** Lollipop charts showed mutation mapping of different genes.** (K)** Tumor mutation was loaded of the high- and low-risk groups. **(L)** The correlation between the TMB and the risk score correlation. **(M)** OS curves for different tumor mutation load and risk score subgroups. **(N)** ROC curves depicted the sensitivity and specificity of the TMB score in predicted 1-, 3-, and 5-year survival in the cohort.** (O)** Violin plots demonstrated the sensitivity of tumor cells in the high- and low-risk groups to different drugs. *P < 0.05, **P < 0.01, ***P < 0.001, ****P < 0.0001. "ns" was used to say that there was no significant difference.

Gene enrichment analysis employs predefined sets of genes and gene ranks to identify significant biological changes or patterns of gene co-expression, and thus to assess functional associations with the set of target genes in the experimental set. In the comparison between high and low-risk groups, a volcano plot of characterized differential genes was demonstrated (Figure 10A). The heatmap, in turn, presented the expression of DEGs in the high-risk and low-risk groups (Figure 10B). The results of the GO analysis showed that DEGs were enriched in several biological processes, including immunoglobulin complex, immunoglobulin complex circulating, external side of plasma membrane, immunoglobulin production, and production of molecular mediator of immune response (Figure 10C). In contrast, the chord diagram of GO enrichment analysis demonstrated the relationship between DEGs and the top 10 most abundant GO pathways (Figure 10D). In addition, KEGG analysis showed that DEGs were enriched in multiple pathways, including Primary immunodeficiency, Hematopoietic cell lineage, B cell receptor signaling pathway, alpha- Linolenic acid metabolism, and Linoleic acid metabolism. The top 20-enriched KEGG pathways are shown in Figure 10E. The signaling pathways associated with the differential genes were evaluated by GSEA analysis. The top 20 signaling pathways showed results indicating that high expression of these DEGs was mainly associated with Mitochondrial Translation, Nucleosome Assembly, Embryonic Digit Morphogenesis, Collagen Fibril Organization, the Snrna Processing, Polyketide Metabolic Process, Protein Hydroxylation, Paraxial Mesoderm Development, Regulation of Gonadotropin Secretion. Response to other pathways related to, in contrast, B Cell Receptor Signaling Pathway, Complement Activation, Immunoglobulin Production, Humoral Immune Response Mediated By Circulating Immunoglobulin, Membrane Invagination, Antigen Receptor Mediated Signaling Pathway, Phagocytosis Recognition Positive Regulation Of B Cell Activation, Production Of Molecula Or Mediator Of Immune Response, Regulation Of B Cell Activation, and other signaling pathways were only enriched for low differential gene expression (Figure 10F). The waterfall plot demonstrated the mutation of the top 30 high mutation frequency genes in 510 samples, with Missense Mutation being the predominant mutation type (Figure 10G). In addition, waterfall plot was also used to demonstrate the mutation status of 14 risk genes, and it was found that only 29 out of 510 patients were mutated, among which the highest mutation rates were found in *FCRL3, ACTB, FCRLA, ZBTB32, PKM* and* MTHFD2*, and the major mutation type was Missense Mutation. (Figure 10H).

The CNV results for the fourteen risk genes were illustrated in Figure 10I. All of these 14 risk genes underwent copy number mutations, including *ACTB, PLD4, GAPDH*. In the TCGA cohort, *ACTB* had Missense_Mutation in four samples, *PCRL3* had Missense_Mutation in five samples and Splice_Site in one sample, *PCRLA* had Missense_Mutation in four samples, the *GABARAPL2* had a Missense_Mutation in two samples, *GAPDH* had a Missense_Mutation in two samples, *ITGB7* had a Missense_Mutation in one sample, *MTHFD2* had a Missense_ Mutation, *PKM* had Missense_Mutation in three samples, *PLD4* had Missense_Mutation in one sample, and *ZBTB32* had Missense_Mutation in two samples and Frame_Shift_Del in one sample (Figure 10J). The tumor mutation burden was higher in the high-risk group compared to the low-risk group (Figure 10K). To deeply explore the correlation between tumor mutation load and risk genes, we found that there was a correlation between tumor mutation load and risk score by correlation analysis between risk score and tumor mutation burden (Figure 10L). We further used tumor mutation load to construct a score, and next, we attempted to further determine the value of this score by predicting the prognosis of patients. We divided the patients into four groups, including High_Risk-High_TMB group, High_Risk-Low_TMB group, Low_Risk-High_TMB group and Low_Risk-Low_TMB group, and we observed that the Low_Risk-Low_TMB group had a significant survival advantage over the High_Risk-High_TMB group had a significant survival advantage, and this difference was statistically significant (Figure 10M). To assess the sensitivity and specificity of the TMB score in terms of 1-, 3-, and 5-year survival of patients, we performed a ROC curve analysis in our cohort, which showed that the predictive accuracy of the TMB score was relatively low (Figure 10N). In addition, in order to investigate the sensitivity of the high-risk and low-risk groups to different drugs, we predicted the drug sensitivity of each patient based on the drug sensitivity data in the GDSC database using the "pRRophetic" R package. The results showed that the drugs ABT.263, AKT.inhibitor.VIII, AZD6244 and BMS.708163 had higher IC50 values in tumor cells in the high-risk group, while the other drugs had higher IC50 values in tumor cells in the low-risk group (Figure 10O).

### *In vitro* experimental validation of JAG1

According to the above findings, the SCORE of the C4 CD86+ Memory B cells subpopulation is a risk factor for the prognosis of patients. In addition, cell interaction analysis showed that C4 CD86+ Memory B cells could act on the JAG1 receptor of malignant cells through CD46.

**Figure 11:**
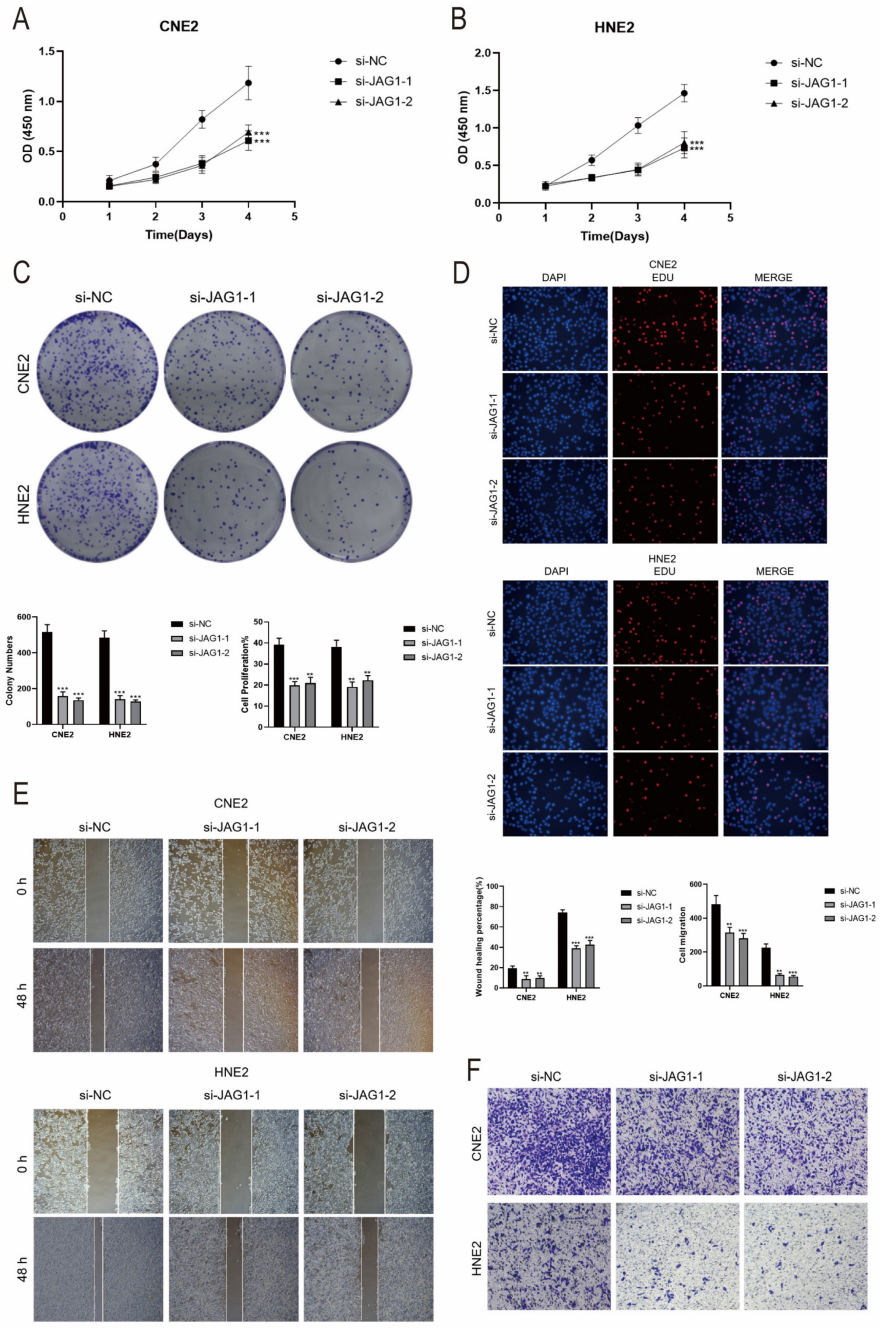
***In vitro* experimental validation of JAG1. (A-B)** CCK-8 assay showed a significant decrease in cell viability after JAG1 knockdown. **(C)** Colony formation assay showed that the colony number of cells in the JAG1 knockdown group was significantly lower than that in the si-NC group. **(D)** EdU staining assay showed that JAG1 knockdown hindered the proliferation of CNE2 and HNE2 cells. **(E)** Scratch assay showed that JAG1 knockdown significantly slowed down the migration of CNE2 and HNE2 cells. **(F)** Transwell assay showed that JAG1 knockdown significantly slowed down the invasion of CNE2 and HNE2 cells. *P < 0.05, **P < 0.01, ***P < 0.001, ****P < 0.0001. "ns" was used to say that there was no significant difference.

Therefore, we hypothesized that C4 CD86+ Memory B cells might be able to promote tumor progression through the CD46-JAG1 pathway. We therefore performed *in vitro* functional experiments to confirm the effects of JAG1 on tumor cells. CCK-8 assay indicated that tumor cell viability was significantly decreased in the JAG1 knockdown group compared with the control group (Figures 11A, B). Meanwhile, colony formation assay and EdU staining assay also showed that cell proliferation was significantly slowed down in both cell lines after JAG1 knockdown (Figures 11C, D). This indicated that JAG1 knockdown could hinder the activity and proliferation of tumor cells while slowing down tumor growth. Next, we confirmed the effect of JAG1 on the migration and invasion ability of tumor cells by scratch assay and Transwell assay. Consistent with the results of previous studies, knockdown of JAG1 also inhibited the migration and invasion of both tumor cells, and the results were statistically significant (Figures 11E, F). JAG1 knockdown can inhibit the proliferation, invasion and migration of NPC cells and inhibit tumor growth.

## Discussion

High-throughput scRNA-Seq is a revolutionary method for cancer research that helps to reveal the heterogeneity among tumors 78. So far, many scientists and doctors have obtained many valuable results related to NPC by this method, exposing the mechanism of NPC development and providing many ideas for NPC treatment and prognosis. NPC is characterized by a large infiltration of lymphocytes, which is significantly associated with tumor progression and the efficacy of immunotherapy 79. Although immune cell subsets other than T cells have not been extensively studied, several reports have concluded that B cells in the TME contribute to the antitumor response 80-83. In patients with NPC, B cells in tertiary lymphoid structures (TLS) can be recruited to improve survival 84. In addition, NPC has a strong etiologic association with EBV infection 85-87. The virus shuttles between B cells and epithelial cells as B cells are the primary host of EBV, which can be transmitted through oral secretions and colonize epithelial cells in the oropharynx 88. Intratumoral B cells have the capability to produce immunoglobulin G (IgG) antibodies, which facilitate the internalization of tumor antigens presented by dendritic cells (DCs), leading to the activation of tumor-reactive T cells. This mechanism is believed to contribute significantly to anti-tumor immunity, with evidence supporting the successful eradication of tumors in murine models 89. In the context of NPC), B cells that infiltrate the tumor generate specific antibodies that mediate their anti-tumor effects through interactions with DCs and T cells 90. Additionally, the expression of TIGIT on memory B cells modulates the immune response by directly interacting with T cells and inhibiting the pro-inflammatory actions of dendritic cells. This results in the suppression of Th1, Th2, Th17, and CXCR5ICOS T cell responses while enhancing the immunoregulatory functions of T cells 91. These findings highlight the significant role of B cells in the progression of NPC.

B cells are key immune cells for anti-tumor immunity and can exert anti-tumor effects through antibody-dependent cytotoxicity and complement activation, and may also be able to promote tumor development and escape.

In this study, based on the CellChat R package model, we reported the signaling ligand-receptor interactions between malignant cells and B cells and inferred the cell-cell communication network in NPC and the roles played by various cells and their significance, some interesting aspects of which are discussed below. Recombinant rabbit monoclonal antibody is a type I transmembrane glycoprotein encoded by the CD99 gene, which is involved in a variety of cellular events such as T-cell recruitment, cellular necrosis of thymocytes and T-lymphocytes, development of pre-B cells, cellular adhesion between lymphocytes, exudation of neutrophils, and wandering of monocytes. Our study found that malignant cells with C9 RGS13+ Germinal center B cells, C8 IGKV1-9+ Naive B cells, C2 ITGB1+ Memory B cells, C4 CD86+ Memory B cells, and C1 NR4A1+ Memory B cells, which are five types of B cells, all of which can act as major senders and receivers of the CD99 signaling pathway, suggesting that the CD99 signaling pathway may play a prominent role in NPC growth and development.

SEMA4 is widely expressed in various tissues and organs of the human body, and through various signaling pathways, SEMA4 plays important biological functions in axon guidance in the nervous system, activation of T and B cells in the immune system and immune regulation. In recent years, a large number of studies have found that SEMA4 is highly expressed in many human tumor tissues and plays an important role in tumor angiogenesis as well as tumor invasion and metastasis, and to some extent, our results are also favorable to the above results. Our study found that malignant cells are the only target cells of SEMA4 signaling pathway, and the sources of SEMA4 ligands are only C1 NR4A1+ Memory B cells and C5 FKBP11+ Plasma cells, so it is speculated that C1 NR4A1+ Memory B cells and C5 FKBP11+ Plasma cells may also be able to promote the development of NPC through the SEMA4 signaling pathway, which provides a highly promising new direction for the treatment of cancer patients, and we can utilize Pepinemab, an antibody targeting SEMA4, to treat NPC patients, so SEMA4 is expected to be used as a target for NPC treatment.

This study identified a risk scoring model based on a composition of 14 risk genes (*FCRL3, FCRLA, ZBTB32, IGFLR1, ITGB7, PLD4, GABARAPL2, ATP6VOE1, ACTB, PKM, GAPDH, VOPP1, TPM3, MTHFD2*), which could be used to predict the overall NPC patients' survival (AUC (1 year) = 0.619, AUC (3 years) = 0.691, AUC (5 years) = 0.664). In our study, one of the novelties was the construction of a predictive model for patients, which was constructed by integrating a variety of different factors, such as age, gender, race, tumor stage, etc., and achieved good predictive accuracy, the area under the ROC curve (AUC) of our model was 0.70 at 1 year, 3 years, and 5 years, respectively, 0.73 and 0.62. It fills the research gap of genetic prognosis prediction model for patients. The findings are expected to provide a theoretical basis for the accurate prognostic assessment of metastatic tumor. Meanwhile, these findings also provide ideas about the mechanism of NPC development. During nasopharyngeal carcinogenesis, tumor-infiltrating lymphocyte B cells (TIL-Bs) in NPC produce IgG in response to various tumor antigens. This production aids in the internalization of dendritic cells that present these antigens, subsequently activating T cells against the tumor cells. In turn, NPC cells attempt to counteract the immune response. Additionally, NPC cells can express several proteins that are either absent or present in minimal amounts in normal nasopharyngeal tissue, which promotes the expansion of myeloid-derived suppressor cells (MDSCs). This expansion adversely impacts B cell differentiation, impairing both B cell maturation and humoral immunity *in vivo* during tumor progression, thereby facilitating the advancement of NPC 90. We can speculate that during nasopharyngeal carcinogenesis, NPC cells can promote the expression of these risk genes to induce MDSC amplification, which directly affects B cell differentiation and makes B cell differentiation and humoral immunity impaired *in vivo* during tumor progression, thus shifting NPC from a low risk to a high risk.

Tumor-infiltrating immune cells play a crucial role in determining tumor progression and aggressiveness, serving as valuable sources of prognostic information for patients. In our study, samples from the low-risk group exhibited higher immune scores and lower tumor purity scores. This suggests that the enhanced immune response and greater immune cell infiltration in the low-risk group compared to the high-risk group may indicate a higher likelihood of benefiting from immunotherapy and achieving a better prognosis. Accurate prognostic models are therefore vital for guiding individualized treatment strategies and evaluating the efficacy of therapies in advanced tumor patients. A robust prognostic model can effectively stratify patients based on their risk of poor outcomes. Additionally, we observed that two types of immune cells—M0 macrophages and resting memory CD4 T cells—had the highest estimated proportions in tumor samples. Conversely, CD8 T cells, plasma cells, naive B cells, regulatory T cells (Tregs), resting mast cells, activated memory CD4 T cells, and macrophages were found in lower estimated proportions in the high-risk group compared to the low-risk group. Notably, M0 macrophages had a higher estimated proportion in the high-risk group, aligning with previous studies that demonstrated a positive correlation between poor prognosis in patients and heightened levels of dendritic cell and macrophage infiltration, alongside a negative correlation with B cells and CD4 T cells 90. For patients classified in the low-risk group, a combination of radiotherapy and immunotherapy emerges as a promising option to enhance treatment outcomes. By integrating detailed insights into immune cell composition, we can better tailor therapeutic approaches, ultimately improving prognostic accuracy and patient management in NPC 92.

Through CellChat analysis, we learned that several B cell subpopulations in NPC can communicate with tumor cells via CD46-JAG1, which is a receptor in the notch signaling pathway that promotes the progression of various tumors, including acute lymphoblastic leukemia, gastric, breast and ovarian cancers, and that inhibition of this pathway can inhibit the progression of many tumors through a variety of pathways. Inhibition of the notch signaling pathway through various channels can inhibit the progression of various tumors. It has been shown that JAG1 is abnormally expressed in various cancers, including acute lymphoblastic leukemia, gastric cancer, breast and ovarian cancer, which affects tumor progression and metastasis, and it has been found that 15D11, a targeting drug for JAG1, can enhance the sensitivity of tumor chemotherapy and reduce bone metastasis, with very mild side effects 93. To investigate the role of CD46-JAG1 signaling pathway in NPC, we performed *in vitro* functional experiments to confirm the effect of JAG1 on NPC tumor cells. The test results showed that knockdown of JAG1 could indeed inhibit tumor invasion as well as migration, and could inhibit tumor progression. Therefore, we speculate that targeting JAG1 may effectively inhibit tumor progression, thereby providing better prognosis for NPC patients. This strategy not only has the potential to enhance treatment outcomes but also to pave new avenues for future immunotherapy. We also recognize several limitations of our study. First, the findings of our predictive model were examined only in a partially open single-cell validation cohort. This limitation suggests that our experimental results may not fully capture the complexity of NPC, and validation in larger cohorts and clinical trials is essential to further elucidate the relevant mechanisms and prognostic therapeutic strategies in NPC tumor progression. Additionally, the efficacy of immune checkpoint inhibitors is often limited, which may be attributed to their specific effects on T-cell-related responses. The composition of NPC also varies considerably between patients, and we are currently only able to confidently characterize the more representative and abundant subtypes in the TME. Minor subpopulations within the NPC microenvironment may significantly influence clinical outcomes, and their identification and characterization remain crucial. Therefore, employing advanced single-cell sequencing technologies to analyze these finer subgroups within NPC tumor masses is necessary for a comprehensive understanding of their roles and impacts.

## Conclusion

In summary, the heterogeneous TME of NPC was revealed by single-cell high-resolution, and through transcriptome analysis of 21526 B cells of 13 subtypes, we identified essential cells and molecules potentially contributing to NPC tumorigenesis, revealing the specificity of the C4 CD86+ Memory B cells subpopulation in NPC. In addition, we combined trajectory analysis to depict the potential developmental trajectories of B cells in the subpopulation of B cells within the tumor. Then, we also obtained the intercellular communication network between B cells and malignant cells using CellChat. Then, we used a large-scale data to construct an immune-related risk model among different expressed genes in different patients, which predicted OS more accurately and was closely related to the level of immune infiltration of cells and the therapeutic efficacy of tumor chemotherapeutic agents. Thus, it provides insights into the mechanism of NPC progression, prediction of NPC prognosis and development of potential therapeutic strategies for NPC.
